# Development of an optogenetic toolkit for neural circuit dissection in squirrel monkeys

**DOI:** 10.1038/s41598-018-24362-7

**Published:** 2018-04-30

**Authors:** Daniel J. O’Shea, Paul Kalanithi, Emily A. Ferenczi, Brian Hsueh, Chandramouli Chandrasekaran, Werapong Goo, Ilka Diester, Charu Ramakrishnan, Matthew T. Kaufman, Stephen I. Ryu, Kristen W. Yeom, Karl Deisseroth, Krishna V. Shenoy

**Affiliations:** 1 Neurosciences Program, Stanford University, Stanford, CA USA; 2 Department of Electrical Engineering, Stanford University, Stanford, CA USA; 3 Department of Neurosurgery, Stanford University, Stanford, CA USA; 4 Department of Bioengineering, Stanford University, Stanford, CA USA; 5 Department of Psychiatry and Behavioral Science, Stanford University, Stanford, CA USA; 6 Department of Neurobiology, Stanford University, Stanford, CA USA; 7 Department of Radiology, Stanford University, Stanford, CA USA; 8 Department of Otophysiologie, Albert Ludwig University of Freiburg, Freiburg im Breisgau, Germany; 9 BrainLinks-BrainTools, Albert Ludwig University of Freiburg, Freiburg im Breisgau, Germany; 10 Cold Spring Harbor Laboratory, Cold Spring Harbor, NY, USA; 11 Palo Alto Medical Foundation, Palo Alto, CA USA; 12 Howard Hughes Medical Institute, Stanford University, Stanford, CA USA

**Keywords:** Motor cortex, Neural circuits

## Abstract

Optogenetic tools have opened a rich experimental landscape for understanding neural function and disease. Here, we present the first validation of eight optogenetic constructs driven by recombinant adeno-associated virus (AAV) vectors and a WGA-Cre based dual injection strategy for projection targeting in a widely-used New World primate model, the common squirrel monkey *Saimiri sciureus*. We observed opsin expression around the local injection site and in axonal projections to downstream regions, as well as transduction to thalamic neurons, resembling expression patterns observed in macaques. Optical stimulation drove strong, reliable excitatory responses in local neural populations for two depolarizing opsins in anesthetized monkeys. Finally, we observed continued, healthy opsin expression for at least one year. These data suggest that optogenetic tools can be readily applied in squirrel monkeys, an important first step in enabling precise, targeted manipulation of neural circuits in these highly trainable, cognitively sophisticated animals. In conjunction with similar approaches in macaques and marmosets, optogenetic manipulation of neural circuits in squirrel monkeys will provide functional, comparative insights into neural circuits which subserve dextrous motor control as well as other adaptive behaviors across the primate lineage. Additionally, development of these tools in squirrel monkeys, a well-established model system for several human neurological diseases, can aid in identifying novel treatment strategies.

## Introduction

In rodents, optogenetic tools and viral approaches to neural circuit targeting have developed tremendously in the past decade, enabling precise, functional manipulation of neural circuitry and highly targeted approaches to therapeutic intervention in neurological disease states. Largely due to the considerable genetic, anatomical, and behavioral differences between humans and rodent models, translating this experimental toolkit for use in nonhuman primates is critical to refining our understanding of and developing treatments for human neurological disease^1,2^. Among primates, the architectural macro-organization of the neocortex has been largely conserved through evolution^3,4^. This shared structure has enabled fruitful neuroanatomical comparisons, in which the differential cognitive, sensory, and motor abilities and behavioral specializations of each species can be correlated with differences in brain structure. Optogenetic tools promise to accelerate these comparative efforts by facilitating precisely-targeted manipulations of critical pathways in the primate brain. These manipulations, in turn, may provide bi-directional insight across primate species into functional similarities and differences in neural organization.

Functional validation of optogenetic tools has been successfully demonstrated in rhesus macaques^5,6^ and marmosets^7^ using AAV vectors, suggesting that achieving AAV-based transfection of opsins in additional primate species might be readily possible. The behavioral consequences of optogenetic manipulation in rhesus have varied in magnitude, with brain region, behavioral readout, opsin construct, and targeting strategy (e.g.^6,8–15^). Collectively, these reports highlight the need for refined viral targeting strategies to facilitate precise, effective optical control in nonhuman primates^16–19^.

**Figure 1:**
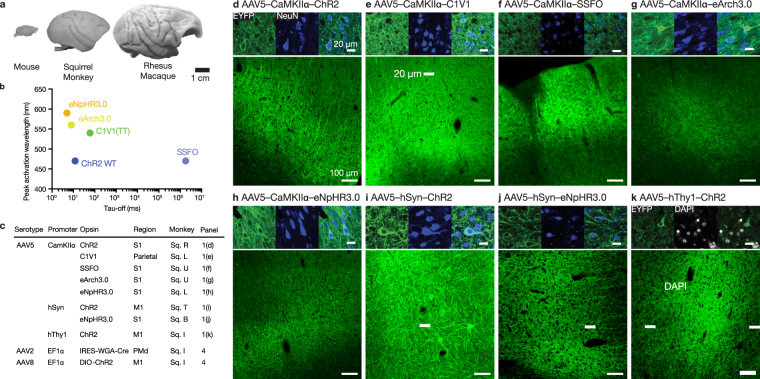
AAV5 successfully transfects cortical neurons in squirrel monkeys. (**a**) Macroscopic image of squirrel monkey brain versus mouse and rhesus macaque brain. (**b**) Opsins were tested across a wide range of opsin deactivation kinetics (*τ*_off_) and activation wavelengths, based on^40,43^. (**c**) Table of opsins successfully transfected, listed by viral serotype, promoter opsin, brain region, and subject identifier. For a full table, see Supp. Table 1. (**d**–**k**) Representative histology for first 8 opsins in table (see Fig. 4 for projection targeting constructs). 40x images show EYFP fluorescence in green, DAPI stain in white or NeuN stain in blue, and merged; scale bars 20 *μ*m. 10x images from coronal slices depict EYFP fluorescence in green at injection site; scale bars 100 μm. We note that in Fig. 1i, the unusually large expressing neurons are Betz cells from layer V of primary motor cortex.

Squirrel monkeys are a versatile, widely used New World primate model in biomedical research^20^. They can be straightforwardly handled and readily trained in a laboratory setting^21^, possess a large brain with a defined central sulcus (Fig. 1a), and display a rich repertoire of social, cognitive, and visually-guided motor behaviors^22–25^. Extending optogenetic tools to squirrel monkeys enables neuroscientists to probe the functional significance of cross-species neuroanatomical differences with fine temporal and anatomical precision, especially when combined with viral approaches capable of targeting particular neural circuit pathways^17,26^.

Here, we report expression of an array of optogenetic tools in squirrel monkeys. First, we demonstrate that a broad panel of depolarizing and hyperpolarizing opsins express in squirrel monkey cortex under the control of multiple genetic promoters (CaMKII*α*, hSyn, and hThy1). Second, we demonstrate high-fidelity optical control even at high evoked firing rates in anesthetized electrophysiological recordings, recapitulating responses reported in rhesus^6,12,18^. Third, we report anterograde and retrograde transduction patterns consistent with trafficking of AAV-based transgenes^16,27,28^. Fourth, we demonstrate for the first time in primates a WGA-Cre dependent dual-viral opsin expression system which enables targeting of cells defined by connections with another brain region for optogenetic manipulation. Fifth, we established that opsins continued to express safely in squirrel monkey cortex for at least one year following viral injection (similar to long-term expression in rhesus macaques), with no adverse health consequences or neurological abnormalities observed. Finally, we established that optogenetic expression patterns, functionality, and long-term viability observed in squirrel monkeys closely resemble observations in rhesus macaques. These similarities underscore the utility of squirrel monkeys as an attractive, complementary model for neural circuit dissection and as a flexible platform for assessing the safety and long-term efficacy of optogenetic manipulations.

## Results

### Local expression of diverse opsin-promoter panel

We injected optogenetic constructs into a total of 12 female squirrel monkeys (mean 13.6 ± 2.6 std. dev. years at time of injection). Supp. Table 1 provides a full listing of injected constructs and locations. We chose to use adeno-associated viral (AAV) vectors to transfect neurons with optogenetic constructs as AAV offers a lower theoretical risk of insertional mutagenesis than lentivirus^29–32^ and has been employed in several human clinical trials^33,34^. For eight constructs, we selected AAV2/5, a pseudotyped AAV that combines the genome of AAV2 with the capsid of AAV5 to improve transduction efficiency and neural tropism (https://www.addgene.org/viral-vectors/aav/aav-guide/). By convention, this pseudotyped AAV2/5 is referred to by its capsid component as AAV5. AAV5 efficiently transduces neurons in both wild-type rats and rhesus monkeys^6,35–38^. We successfully transduced both depolarizing (ChR2(H134R), C1V1(TT), SSFO) and hyperpolarizing (eNpHR3.0, eArch3.0) opsins^39–43^, chosen to span a large range of opsin deactivation kinetics (*τ*_off_) and activation wavelengths (Fig. 1b)^40,43^. This range of constructs enables genetically-targeted, bidirectional optical control (Fig. 1c) and were expressed in neurons across an array of cortical regions at 6–12 weeks post-injection (Fig. 1d–k, Supp. Fig. 1d to k). Strong expression was reliably observed within 1 mm of the injection site, although we anticipate that the exact viral spread will depend on viral titer and the tropism and expression strength of the particular serotype and promoter combination selected. Minimal gliosis was observed outside of the immediate vicinity (50 μm) of the injection site (Supp. Fig. 2).

### Expression in axonal projections and in connected neurons

**Figure 2:**
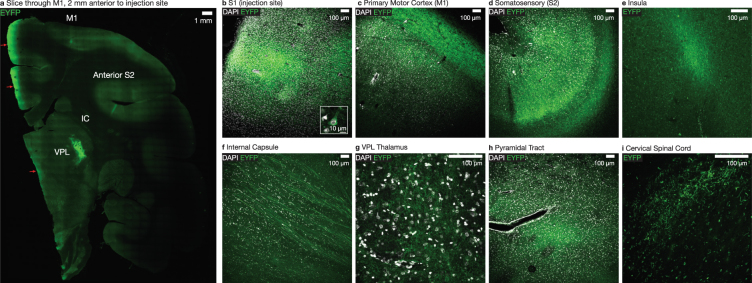
eNpHR3.0 expresses in fibers in downstream brain circuitry, enabling circuit-specific neural modulation. (**a**) AAV5-hSyn-eNpHR3.0-EYFP expression in M1 coronal slice, 4 mm anterior to the injection site in left S1 of Sq. B, indicating expression in downstream projection targets. Red arrows indicate regions where tissue brightness is presumed to be due to aberrations in mounting at the edges of the large slice. (**b**) eNpHR3.0 expression near the injection site in S1. Inset shows representative expressing cell body. (**c**–**i**) Opsin expression in fibers in brain regions downstream of S1, including in M1 cortex, anterior secondary somatosensory cortex (S2), insula, internal capsule, VPL thalamus, the pyramidal tract at the medulla of the brainstem, and corticospinal tract imaged in the posterior (dorsal) column of the cervical spinal cord.

In contrast to more genetically tractable rodent models in which transgenic lines and specific promoters are prevalent^40,44,45^, strategies for genetically-targeted optical control in primates are less well-developed. However, many fundamental questions in systems neuroscience concern the interactions between brain areas of interest. These questions could be tackled experimentally by targeting optogenetic constructs or other transgenes to anatomically-defined projections. For example, by optically inhibiting axonal terminals in primary motor cortex (M1) which arise in primary somatosensory cortex (S1), one could potentially optically suppress sensory input to motor cortex during reaching movements and explore the neural and behavioral consequences of this manipulation. Towards this end we evaluated whether the expression patterns of opsins injected in squirrel monkey cortex would enable optogenetic manipulation targeted to specific projections. In a representative squirrel monkey (Sq. B), injected with AAV5-hSyn-eNpHr3.0-eYFP in primary somatosensory cortex (S1) (Fig. 2b), we observed eYFP fluorescence in axonal projections in downstream primary motor cortex (M1) (Fig. 2c), internal capsule (Fig. 2f), the ventral posterolateral nucleus (VPL) of thalamus (Fig. 2g), the pyramidal tract (Fig. 2h), and in cervical spinal cord (Fig. 2i). These findings are consistent with the established use of AAV infection for anterograde tracing^46^. Importantly, this robust axonal expression suggests that restricting light delivery to a downstream target could enable optical modulation of projection terminals, facilitating optical control over information flow between brain regions of interest.

**Figure 3:**
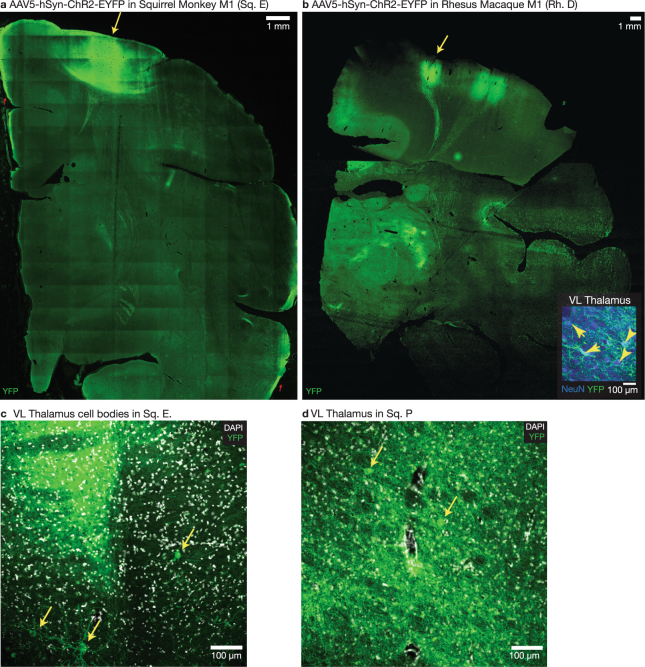
Retrograde transduction in both squirrel monkey and rhesus macaque. In both squirrel monkey and rhesus macaque, AAV5 expresses in cell bodies in the ventral lateral (VL) nucleus of the thalamus, suggesting viral uptake and retrograde transduction in thalamocortical axons. (**a**) AAV5-hSyn-ChR2(H134R)-EYFP injected in Sq. E left M1 at the site indicated by yellow arrow. Autofluorescence along the midline and edges of the tissue, indicated with red arrows, is due to aberrations in slide mounting rather than opsin expression. (**b**) AAV5-hSyn-ChR2(H134R)-EYFP injected in Rh. D in right M1 at the site highlighted by yellow arrow. Inset: Expression in cell bodies in VL thalamus, highlighted with yellow arrows. (**c**) Expression in cell bodies in VL thalamus of Sq. E, highlighted with yellow arrows. (**d**) Expression in cell bodies in VL thalamus of Sq. P, also injected with AAV5-hSyn-ChR2(H134R)-EYFP in left M1.

We also observed transduction of AAV5 along corticothalamic projections. In squirrel monkeys Sq. E and Sq. P, following injection of AAV5-hSyn-ChR2(H134R)-eYFP into M1 (Fig. 3a) we observed expression in cell bodies in upstream ventral lateral (VL) thalamus which relays cerebellar inputs to M1 (Fig. 3c,d). This suggests either retrograde transduction–AAV5 was taken up by thalamocortical axonal projections and trafficked to thalamic cell bodies–or anterograde, transsynaptic tranduction–AAV5 was transported along corticothalamic axons, released at terminals, and taken up by connected thalamic neurons. We also observed expression in processes in VL, likely in both projections from M1 to VL and the processes of the expressing VL cell bodies. In macaques, we observed a similar corticothalamic transduction pattern with expressing cell bodies in VL thalamus following the injection of the same construct into M1 of macaque Rh. D. (Fig. 3b). This comparison highlights the microstructural similarities between rhesus squirrel monkey anatomy and viral transfection patterns.

### Dual-injection strategy for targeting circuit projections

Axonal expression and transduction may be useful for certain circuit manipulations, but we sought to achieve more precise targeting of anatomically defined circuit elements using a combinatorial dual-viral injection approach. Additionally, such an approach might prove useful in targeting cortico-cortical projection neurons that resist transfection by retrograde transduction or anterograde, transcellular transfer via a single AAV construct (see Discussion).

We first attempted to utilize long-term herpes simplex virus (LT-HSV) to target anatomically-defined projections. In Sq. R., we injected LT-HSV-Ef1α-Cre-mCherry^47^ into M1 and AAV5-hSyn-DIO-eYFP into PMd. However, we observed significant neuronal cell death near the M1 injection site (Supp. Fig. 3).

**Figure 4:**
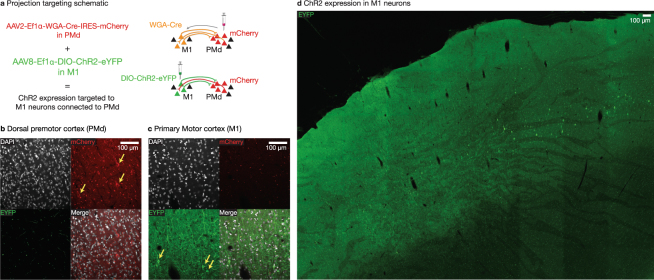
WGA-Cre enables targeting projections for circuit-specific neural modulation. (**a**) Schematic illustrating injection of AAV2-Ef1*α*-mCherry-IRES-WGA-Cre in PMd and AAV8-Ef1*α*-DIO-ChR2(H134R)-EYFP in M1 in order to target M1 cells that are connected to PMd. (**b**) Right PMd cell bodies expressing mCherry and projecting fibers expressing ChR2(H134R)-EYFP in Sq. I. (**c**) Right M1 cell bodies highlighted with yellow arrows expressing ChR2(H134R)-EYFP in Sq. I. (**d**) Large overview image of EYFP-ChR2(H134R) expressing neurons in M1.

In another squirrel monkey Sq. I, we employed an alternative targeting strategy (Fig. 4a). We injected the an AAV2 viral vector containing transsynaptic tracer wheat-germ agglutinin (WGA) fused to Cre-recombinase with an mCherry reporter in dorsal premotor cortex (PMd) (AAV2-Ef1*α*-mCherry-IRES-WGA-Cre). This resulted in expression of mCherry in PMd (Fig. 4b, 50 mCherry^+^/199 DAPI^+^) and transsynaptic transport of WGA-Cre, expressed independently of mCherry due to IRES, to connected cell bodies in M1^40,41^. We then injected a Cre-dependent ChR2(H134R) construct (AAV8-Ef1*α*-DIO-ChR2(H134R)-eYFP) into M1. This configuration was designed to target expression of ChR2(H134R) to the anatomically-defined subset of M1 neurons connected with PMd. This approach resulted in ChR2(H134R) expression in M1 neurons (Fig. 4c, 7 YFP^+^/71 DAPI^+^) which putatively project to or receive projections from PMd. Near the injection sites, neuronal morphology based on YFP staining suggested that neurons remained intact and without gross evidence of tissue destruction. Consistent with restricted spread of WGA-Cre from PMd to M1, we observed no mCherry-expressing neurons in the vicinity of the M1 injection site (0 mCherry^+^/71 DAPI^+^) and no eYFP-expressing neurons near the PMd injection site (0 YFP^+^/199 DAPI^+^), although see Discussion for potential caveats. To our knowledge, this represents the first validation of a dual-viral, transsynaptic strategy for targeting opsin expression to projection targets in primates, an approach which facilitates a new class of experiments utilizing precise anatomically-specific optical control.

### Opsin functionality

**Figure 5:**
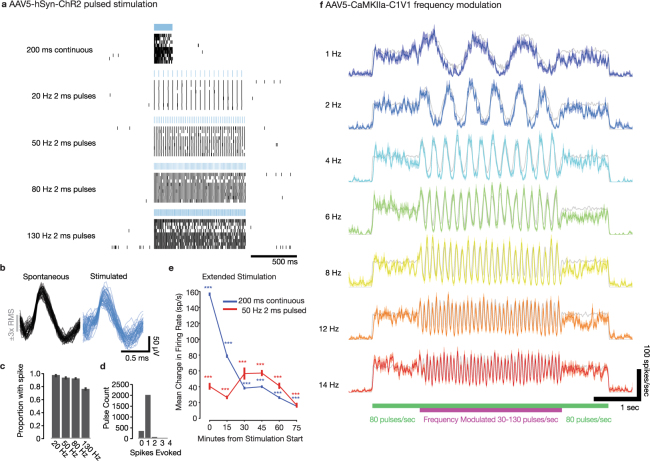
*In vivo* optogenetic stimulation and electrophysiology facilitates verification of optogenetic modulation in an anesthetized squirrel monkey. (**a**) Spike response raster during pulsed and continuous stimulation with 473 nm light of an example recorded single-unit recorded in a Sq. P with AAV5-hSyn-ChR2(H134R) in left M1. (**b**) Spontaneous and light-evoked spike waveforms. (**c**) Proportion of light pulses which evoked at least one spike in this unit vs. pulse frequency. Error bars show 95% confidence intervals for success probability (Clopper-Pearson method). (**d**) Histogram of spike counts evoked per light pulse. (**e**) Optogenetic response to extended stimulation over 90 minute stimulation session. Change in firing rate (stimulation minus per-trial pre-stimulation baseline) of recorded multiunit activity evoked by optical stimulation with 200 ms continuous pulses delivered at 1 Hz (in Sq. E left M1), or 50 Hz 2 ms pulse trains delivered for 5 sec interspersed with 10 sec off periods (in Sq. T, left M1). Error bars show ± SEM. “***” indicates significant elevation of firing rates by stimulation during the corresponding 15 minute epoch (*p* < 0.001, two-sided sign-rank test). (**f**) Light-evoked firing rate responses of an example AAV5-CamKII*α*-C1V1-transfected neuron recorded in Sq. U left PMd, during frequency-modulated pulsed stimulation with 561 nm light. Gray lines indicate the idealized average firing rate that would be evoked if the unit spiked exactly once per light pulse. Color shading indicates ± SEM. See Methods.

To verify the functionality of the optogenetic constructs, we developed a mobile optical stimulation and electrophysiology cart (Supp. Fig. 4) in order to perform anesthetized recordings in previously injected squirrel monkeys in the operating room. In a squirrel monkey (Sq. P) injected with AAV5-hSyn-ChR2(H134R), well isolated single-units (Fig. 5a–d and Supp. Fig. 5) and multi-unit activity (Supp. Figs 6 and 7) responded robustly to optical stimulation at 473 nm. In contrast, 635 nm wavelength light pulses did not evoke detectable neural responses (Supp. Fig. 8), as expected from the opsin’s activation spectrum. Both pulsed and continuous stimulation reliably evoked spiking activity at the optrode (Fig. 5c,d, Supp. Fig. 5b).

In another squirrel monkey (Sq. U), we injected the red-shifted opsin, AAV5-CamKII*α*-C1V1 into PMd. Near the injection site, 561 nm light drove reliable responses in multiunit activity (Supp. Fig. 9), as expected from this opsin’s activation spectrum and previously demonstrated in rodents^48^. Opsins such as C1V1 with red-shifted activation spectra are important for species with larger brain volume, as the reduction in light scattering in brain tissue with longer wavelengths of light may permit greater depth of penetration^40^.

We next designed a stimulation protocol to test the fidelity of optogenetic control in our anesthetized preparation over longer timescales common in rhesus awake-behaving neurophysiology experiments. Over 90 minutes of episodic stimulation, we tracked the response of multi-unit activity in two squirrel monkeys (Sq. E and Sq. T) injected with AAV5-hSyn-ChR2(H134R) in M1 cortex (Supp. Figs 10 and 11). Pulse trains (50 Hz) and continuous pulses (200 ms) both elevated firing rates for the full 90 minute period (*p* < 0.001, sign-rank test), although pulse trains evoked more consistent responses over the full duration (*p* < 0.001, rank-sum test).

Many neurological conditions implicate abnormal oscillatory rhythms in or across specific brain regions, suggesting that the ability to drive rhythmic patterns using optogenetic tools may be useful for future therapies^28,49^. We explored this possibility in an anesthetized squirrel monkey (Sq. U) injected in M1 with AAV5-CaMKII*α*-C1V1(T/T), an opsin with slightly slower kinematics and enhanced light sensitivity relative to ChR2(H134R)^48^. We delivered a frequency-modulated optical stimulus. The laser delivered constant-amplitude light pulses at a “carrier” rate that was varied between 30 Hz and 130 Hz. The carrier rate was varied according to a sinusoidal “signal” pattern at modulation frequencies up to 14 cycles/sec. This stimulation paradigm reliably evoked rapid rhythmic modulation of nearby firing rates according to the signal pattern (Fig. 5f).

### Long-term opsin expression and animal health

**Figure 6:**
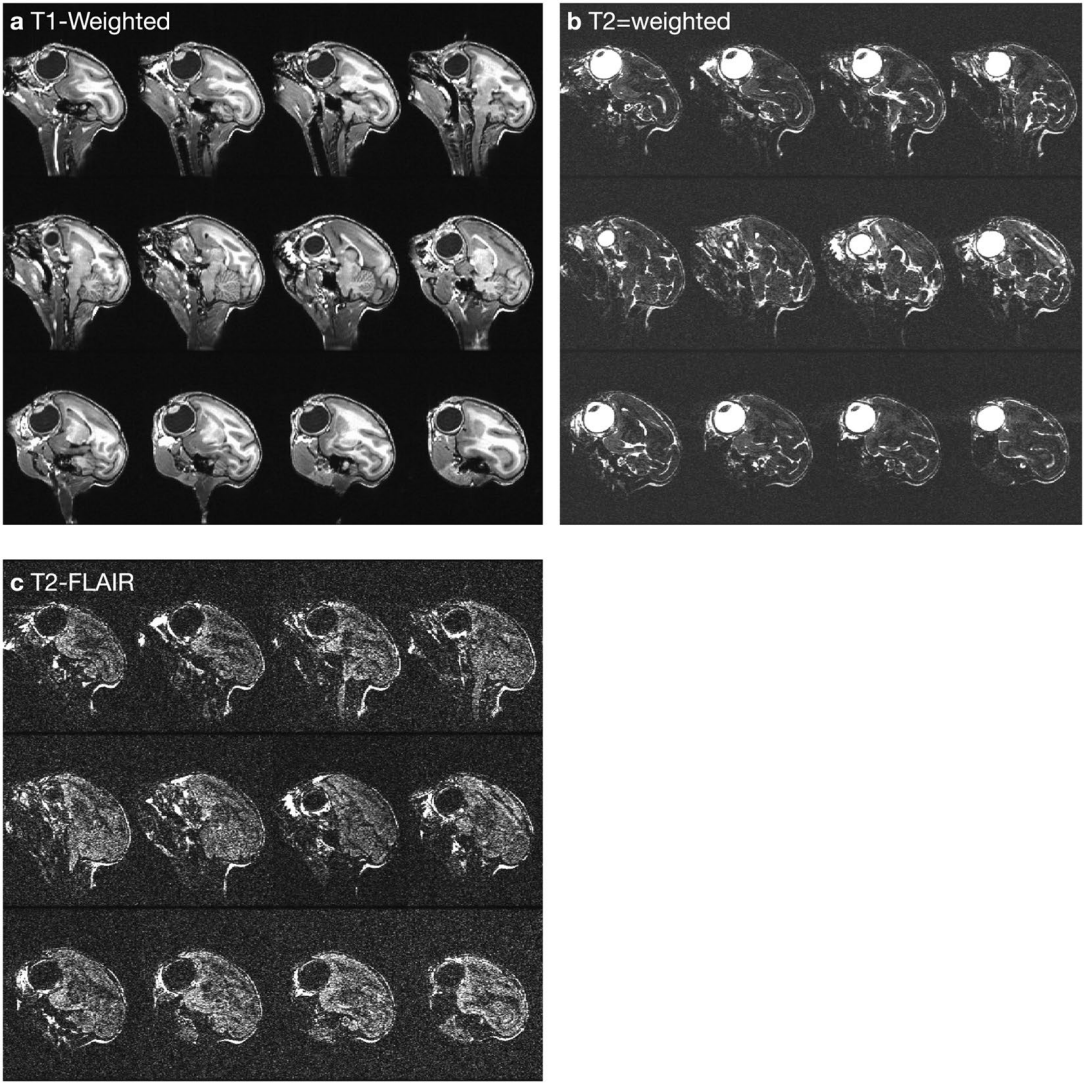
Long-term sagittal MR images show normal brain morphology without pathologic changes in Sq. M at one-year post injection of AAV5-hThy1-eNpHR3.0-EYFP in Left M1 and AAV5-CaMKII*α*-eNpHR3.0-EYFP in Right S1. Three types of coronal scans were acquired per monkey and reformatted in the sagittal plane for visualization: (**a**) T1-weighted 3D IR-FSPGR sequence which maximizes gray-white matter contrast, (**b**) T2-weighted fast spin echo (FSE), and (**c**) T2-weighted fluid-attenuated inversion recovery (FLAIR) sequences which are used to identify potential brain pathology. Upon review by a clinical neuroradiologist, the scans did not reveal any pathologic changes. Specifically, no imaging features of injury, hemorrhage, edema, demyelination, or inflammatory changes were present.

As neuroscience experiments in primates often require long time periods of behavioral training, it is critical to validate that virally-delivered optogenetic constructs remain safe and effective in primates over long timescales. Towards this end, we utilized the squirrel monkey model to establish whether virally-transfected optogenetic constructs continue to express over long timescales and whether such long-term expression causes any adverse neurological consequences. We injected four squirrel monkeys with either AAV5-hThy1-ChR2(H134R)-eYFP (Sq. D and Sq. H) or AAV5-hThy1-eNpHR3.0-eYFP (Sq. M and Sq. O) in M1. To assess the safety of long-term opsin expression, we monitored the health of all four squirrel monkeys for one year. During this time period, the squirrel monkeys were overall alert, active, and healthy, and no adverse health effects were noted. We also performed a standard panel of blood tests on each squirrel monkey at timepoints before and after one year post-injection (Supp. Table 2). These results confirmed the squirrel monkeys remained in good health with no detectable inflammatory response, consistent with undetectable levels of TNF*α* and C-reactive protein in the blood. We also acquired T1-weighted, T2-weighted, and T2-FLAIR brain MRI images of Sq. M (Fig. 6 and Supp. Fig. 13) and Sq. O (Supp. Figs 14 and 15) at one year post-injection. These images, reviewed by a clinical neuroradiologist, did not exhibit any pathological features (e.g. focal parenchymal injury, hemorrhage, demyelination, edema, or features that would suggest inflammation).

**Figure 7:**
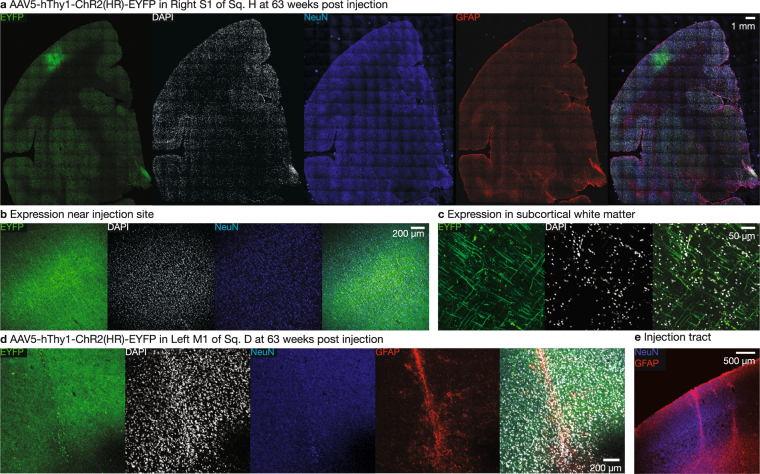
Long-term opsin expression in squirrel monkeys. (**a**) Expression of AAV5-hThy1-ChR2(H134R)-EYFP at 63 weeks post-injection in Sq. H in half coronal slice located near injection site in left M1. Low GFAP expression suggests minimal gliosis. (**b**) Expression in cell bodies of neurons located near injection site and (**c**) in crossing axonal fibers in subcortical white matter. (**d**) Expression of AAV5-hThy1-ChR2(H134R)-EYFP at 63 weeks post-injection in Sq. D left M1. (**e**) Gliosis was limited to the immediate vicinity of the injection track.

After at least one year post-injection, we assessed opsin expression in Sq. D and Sq. H. In both monkeys, strong expression was visible in the vicinity of the injection site (Fig. 7a,b,d) as well as in projecting fibers in subcortical white matter (Fig. 7c). We observed minimal gliosis limited to the immediate vicinity of the injection track (Fig. 7d,e), similar to our results in the short-term expression monkeys (Supp. Fig. 2).

**Figure 8:**
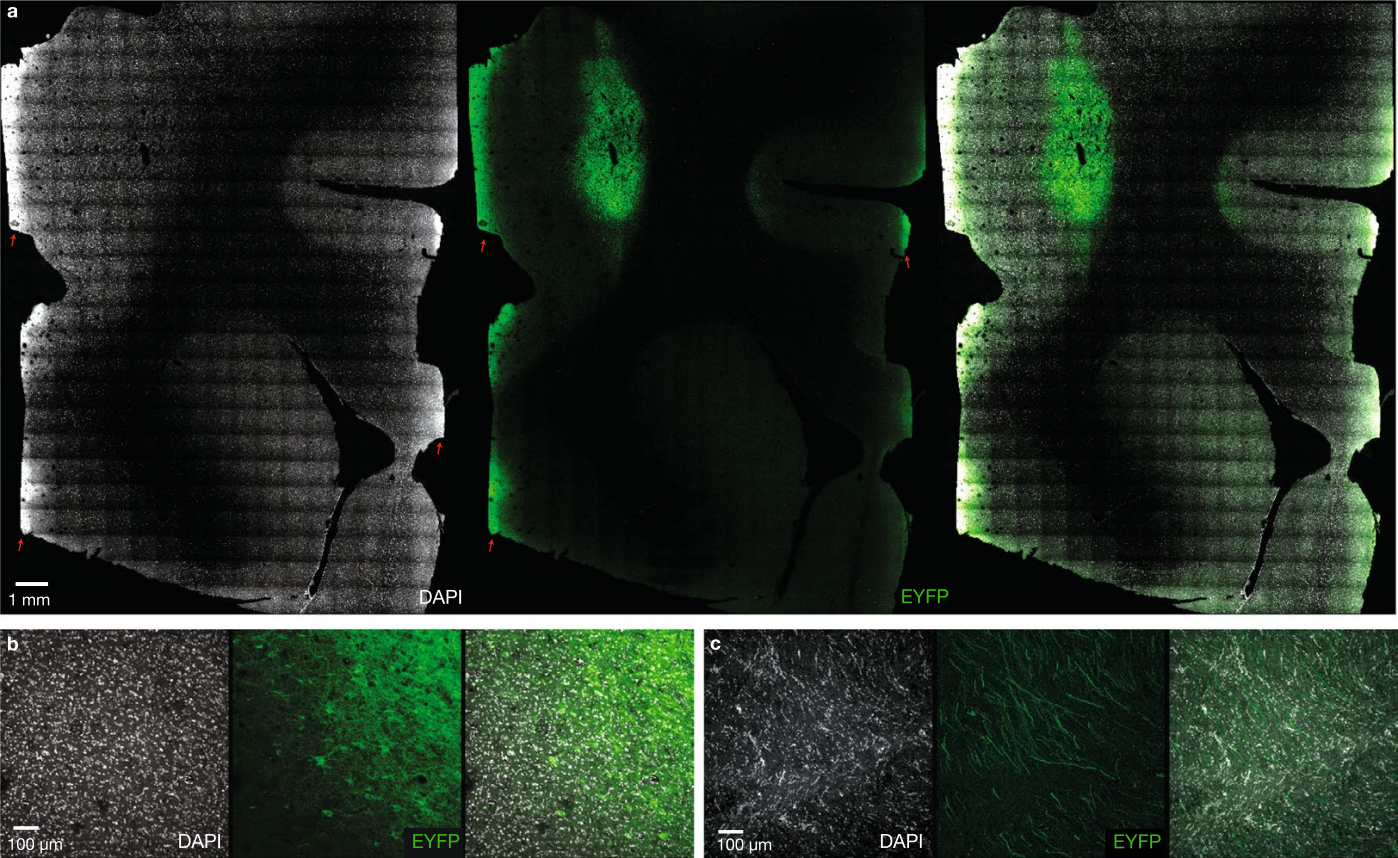
Long-term opsin expression in a rhesus monkey. (**a**) Expression of AAV5-CaMKII*α*-C1V1(TT)-EYFP injected into left PMd at 188 weeks post-injection in Rh. Q. Autofluorescence along the midline and edges of the tissue, indicated with red arrows, is due to aberrations in slide mounting rather than opsin expression. Tissue damage near the injection site is presumed to be the result of repeated optrode and electrode penetrations. (**b**) Expression was seen in cell bodies of neurons located in the vicinity of the injection site and (**c**) in crossing axonal fibers in subcortical white matter.

To further validate the long-term efficacy of optogenetic expression, we performed a similar experiment in 2 rhesus macaques (Rh. O and Rh. Q). These macaques were injected with AAV5-CaMKII*α*-C1V1(TT)-eYFP in either PMd (Rh. Q) or both M1 and PMd (Rh. O). At 188 weeks post-initial injection, we performed histology on rhesus monkey Q. These images demonstrated strong expression in the vicinity of the C1V1 injection sites (Fig. 8a,b) and in subcortical white matter below the injections (Fig. 8c). To establish the long-term functionality of the C1V1 opsin, we performed repeated optrode stimulation and recording sessions from 130–240 days from injections. For each unit or multi-unit recorded at the optrode, we measured the evoked changes in firing rates while the monkey was waiting passively between trials of a reaching task. Strong responses were detectable throughout the entire time period measured (Supp. Fig. 16), and only very small trends towards reduced efficacy were observed in both rhesus monkeys, though neither reached statistical significance (Rhesus Q: −0.49 spikes/sec/day [−1.08 to 0.10, 95% CI], n = 78 units; Rhesus O: −0.11 spikes/sec/day [−0.35 to 0.13, 95% CI], n = 74 units).

## Discussion

The goal of this project was to demonstrate successful transfection of optogenetic constructs in squirrel monkeys. Here, we demonstrate the ability to assess diverse opsin panels in the squirrel monkey brain, functional validation of optically-evoked responses *in vivo*, strategies to target optical control to specific neuron types or projection targets, and the feasibility of long-term experiments in which continued opsin expression and animal health are maintained over the long timescales characteristic of nonhuman primate research. AAV5 successfully transfected cortical neurons and enabled expression of a variety of inhibitory (eNpHR3.0 and eArch3.0) and excitatory (ChR2(H134R), C1V1(TT) and SSFO) constructs under the control of the CaMKII*α*, hSyn, and hThy1 promoters. As discussed below, histology revealed expression of the panel of promoter and opsin combinations across 12 squirrel monkeys that closely paralleled opsin expression patterns and functionality in macaques, including anterograde trafficking to known downstream projection targets and retrograde transduction along thalamocortical inputs. We note that all of our injections were in an older cohort of female squirrel monkeys; we expect that expression might be stronger in younger animals. While we anticipate that our results should generalize to male squirrel monkeys, further experimentation would be required to verify similar expression patterns.

### Targeting of projection pathways

#### Anterograde spread

In our panel of squirrel monkeys injected with AAV5, histology demonstrated opsin expression in axonal projections originating at the cortical injection sites and terminating in known downstream targets, consistent with well-established anterograde expression patterns noted with recombinant AAV vectors [e.g.^46^]. This presents the possibility of delivering light at distal axonal terminals to selectively activate or suppress specific circuit outputs, as has been effectively employed in rodents (e.g.^50–52^) and macaques (e.g.^14,16^). The strength of anterograde expression in axonal projection as well as the spread of expression around the injection site are likely dependent on both the overall strength of transgene expression (in turn modulated by the injection parameters, viral titer, viral tropism, promoter strength, etc.) as well as the trafficking of the transgene along axonal fibers. This transport efficiency may be enhanced by using specific post-transcriptional targeting sequences that result in improved membrane targeting of the opsin protein^41,43^. While the overall efficiency and tropism of AAV1, 2, 5, 8, and 9 have been compared in primates^7,38,53^, more systematic, quantitative comparisons are needed.

#### Retrograde and anterograde transduction

In both macaques and squirrel monkeys, we also observed expression in cell bodies located in the ventral lateral (VL) nucleus of the thalamus following injection of AAV5-hSyn-ChR2(H134R) in primary motor cortex (M1). These expressing VL neurons could have been transduced via either retrograde or anterograde transduction, which are difficult to dissociate in this circuit due to the presence of reciprocal connections between M1 and VL^54^. In retrograde transduction, the virus would infect VL axon terminal fields in M1 and be transported back to cell bodies in VL. In anterograde, transsynaptic transduction, the virus would be transported along M1 axons terminating in VL, released at the axon terminal, and be taken up by second order VL cell bodies. The relative propensity for transsynaptic anterograde versus retrograde transduction of adeno-associated viruses is known to be serotype dependent^55^. Transsynaptic, anterograde transduction has been previously demonstrated with AAV2 in rats^56^ along nigrostriatal projections and in macaques along nigrostriatal and thalamocortical projections^57,58^, and with pseudotypes AAV2/8 and 2/9 in mice from entorhinal cortex to dentate gyrus^59^.

Retrograde transduction has also been previously demonstrated with recombinant AAV vectors both in rodents and primates. In mice, recombinant AAV pseudotypes 2/1, 2/5, 2/7, and to lesser extent 2/8 all drive retrograde transduction along nigrostriatal projections^35^. AAV2 did not result in retrograde expression in this context but is capable of weak transduction from skeletal muscles back to motor neurons in the spinal cord^60^ and between the hippocampi^61^. AAV2/1 injected in S1 also retrogradely transfects layer 5 pyramidal neurons in the contralateral S1^62^. However, Rothermel and Brunert^62^ reported dramatic differences in the ability of rAAV constructs with identical pseudotype to mediate retrograde expression solely attributed to differences in the vector construct itself. In rats, pseudotypes AAV1, 2/1 and 2/5 (but not 2/2) exhibit retrograde transduction along nigrostriatal projections^63,64^ and AAV2/1 transduces medial entorhinal cortical neurons retrogradely from the hippocampus^65^. In primates, retrograde transduction has been reported with multiple AAV serotypes along several axonal pathways. AAV1 mediates nigrostriatal transfection in cynomolgus macaques^64^. AAV6 mediates retrograde transduction from the gastrocnemius muscle to lumbar motor neurons in African green monkeys^66^ and along corticostriatal, nigrostriatal, and thalamostriatal axonal pathways in cynomolgus macaques^67^. AAV8 and AAV9 injections in striatum labeled nigrostriatal, corticostriatal, thalamostriatal and geniculocortical pathways in marmosets^68^.

Collectively, this literature suggests that anterograde and retrograde transduction by recombinant AAVs likely depends on viral serotype, the viral vector construct, and the identity of the axonal projection. It is also possible that expression time, viral titer, injection protocol, and the genetic promoter may modulate the efficiency of retrograde transduction at terminals and/or of anterograde transduction and uptake by second order neurons. One of these factors may explain the discrepancy between the expression observed here in thalamocortical projection neurons and the results of Galvan and colleagues^12^, who report no expression in motor thalamus following injection in primary motor cortex of either AAV5-CaMKII*α*-ChR2(H134R) or AAV5-CaMKII*α*-C1V1. The difference in promoter is notable, although thalamic projection neurons would be expected to express CaMKII*α* as well^69^.

Remarkably, we did not observe transduction of AAV5 from primary motor cortex along any of the known corticocortical connections with posterior parietal, primary somatosensory (1,2,3a), and frontal regions (notably ventral and dorsal premotor, supplementary motor, and cingulate motor areas)^70–75^. To our knowledge, prior reports of cortico-cortical transsynaptic transduction with AAV are limited to inter-hemispheric spread to contralateral S1, observed weakly with AAV9 in marmosets^7^ and with AAV2/1 in mice^62^. Furthermore^62^, observe that retrograde transfer from V1 injection of AAV8 and AAV9 is limited to the LGN; none of the cortical regions that reciprocally connect with V1 were labeled. Further experiments are needed to explore the conditions in which retrograde gene transfer along cortio-cortical pathways occurs, though we speculate that some differentiating feature of thalamocortical axons^76^ (or nigrostriatal, corticostriatal, thalamostriatal, geniculocortical, etc.) makes them particularly susceptible to AAV infection and retrograde transfer. AAV is internalized by cells via receptor-mediated endocytosis from clathrin-coated pits^77^. The particular receptors and relative efficiencies vary by serotype, and many viruses possess redundant methods for cellular entry that may be preferentially used in certain regions^63,78^.

#### WGA-mediated transduction

In addition to anterograde and retrograde transfer with single constructs, we also demonstrate a dual-viral strategy for restricting opsin expression to the anatomically-defined subset of neurons in one brain region connected with a second brain region, enabling precise targeting of neural circuit projections in the primate brain. Our approach, previously demonstrated in rodents^41,65^, leverages wheat germ agglutinin (WGA) as a transsynaptic tracer to deliver Cre-recombinase to connected neurons which have been infected with a Cre-dependent opsin. Although this method is a promising approach for improving anatomic specificity of opsin expression, we note that there are several possible mechanisms mediating expression of ChR2-eYFP in M1 neurons connected to PMd. Two of these mechanisms leverage WGA as a transsynaptic tracer. One notable feature of using WGA as the transsynaptic tracer is its ability to undergo both anterograde and retrograde transceullar transfer^79,80^. Consequently, AAV2-WGA-Cre-IRES-mCherry (here on referred to as AAV2 for simplicity) could infect PMd neurons, resulting in mCherry expression in cell bodies in PMd. Then WGA-Cre could traffic transsynaptically to presynaptic M1 neurons, leading to recombination and ChR2-eYFP expression in AAV8-DIO-ChR2-eYFP (here on referred to as AAV8 for simplicity) infected M1 neurons that project to PMd. Alternatively, AAV2 could infect PMd neurons and WGA-Cre could first transport anterogradely along the entire length of M1-projecting axons and then travel transsynaptically to connected neurons in M1. Recombination by Cre would then drive ChR2-eYFP expression in AAV8 infected M1 neurons receiving input from PMd. These two possibilities are difficult to distinguish due to reciprocal connectivity between PMd and M1. If this possibility of bi-directional transfer of the recombinase complicates interpretation of a particular optogenetics experiment, a non-toxic fragment of tetanus toxin (TTC) is a potential alternative tracer element which specifically exhibits retrograde transsynaptic transfer in mice^81^.

Two additional possibilities exist which would not leverage WGA to achieve transsynaptic spread. AAV2 could infect M1 neurons at their axonal terminals in PMd and then be transported retrogradely back to the nuclei, leading to recombination and ChR2-eYFP expression if these neurons were co-infected by AAV8. Alternatively, AAV2 could infect PMd neurons and the entire AAV2 particle be transported anterogradely and released at axon terminals and taken up across the synapse by M1 neurons. This mode of anterograde transsynaptic transport has been observed for AAV2 in rats in nigrostriatal pathways^56^. However, as discussed above, reports of transsynaptic transduction via AAV directly (not mediated by WGA) are rare for cortico-cortical connections. Nevertheless, due to recombination, even a small amount of transduction of Cre could drive potentially expression of the opsin in M1 neurons, even if the mCherry reporter expression were too weak to generate a visible signal. More sensitive techniques (e.g. RNA FISH to detect mCherry transcripts) could be used to detect this means of transduction, which would result in different anatomic specificity. In these cases, both promoters would conjunctively gate expression in the dually transfected M1 neurons, as opposed to one promoter gating expression in M1 neurons and the other expression in connected PMd neurons.

Dual-viral strategies like this enable new classes of experiments which require precise manipulation of specific circuit pathways^27^. Moreover, these methods can be combined with cell-type specific promoters to achieve additional precision by restricting expression to the intersection of multiple genetically and anatomically defined cell types^82,83^. Of course, a caveat of this approach is that transsynaptic transport may vary across neural circuits; therefore this dual-vector targeting approach should be validated for each experimental system in which it is employed^40^. Our results validate the use of WGA-Cre to drive opsin expression in reciprocal M1 projections to premotor cortex, which can be used to probe the functional role of these feedback pathways during the preparation and execution of reaching movements.

Precise targeting of specific anatomically defined circuit elements is particularly important for primate neuroscience, where fewer cell-type specific promoter sequences have been established^16^. Projection targeting strategies can enable researchers to dissect neural computations into functional neural circuit components and to establish the causal contribution of different anatomical pathways to complex behavior. Projection targeting approaches may also prove particularly effective in modulating behavior in circuits^14^ where broad, regional modulation has proven less effective^19,84–86^.

We were not successful in one attempt to use LT-HSV to deliver Cre transsynaptically to facilitate expression of a Cre-dependent opsin transgene. Others have reported successful viral transfection over short (<14 days) timescales using LT-HSV in rhesus striatum^87^, suggesting that our results may relate to a number of factors including the duration of expression, the viral titer, or the injection protocol, or may be specific to LT-HSV use in neocortex or in squirrel monkeys. In such circumstances, alternative retrograde viruses such as recombinant pseudorabies virus (PRV)^88^ or helper-dependent canine adenovirus (CAV-2)^89^ could be explored as an alternative to LT-HSV, for certain situations and expression timelines.

### High-fidelity optically-evoked spiking

To test the functionality of expressed opsins, we developed a portable electrophysiology cart to optically stimulate and electrically record single neurons in anesthetized squirrel monkeys in an operating room. Using this system, we confirmed that optical stimulation *in vivo* robustly drove spiking responses in neurons transfected with ChR2(H134R) and C1V1(TT). We show that single neuron and multiunit spiking activity can be reliably evoked optically. We also found that the multiunit firing rate in squirrel monkeys can track sinusoidally modulated “carrier” signals. The ability to amplify or suppress endogenous oscillatory rhythms may prove therapeutically useful for treating certain neurological disorders^49^ and might shed insight on the computational roles of oscillatory dynamics (e.g.^90^).

Optically-evoked responses could be sustained for at least 90 minutes during repeated stimulation with continuous and pulsed light. Furthermore, the responses closely resembled light-evoked responses in optogenetically transfected macaque cortical neurons^6^. It has been noted previously that firing rates in some brain regions may exhibit substantially lower firing rates in rodents than in primates^91^. The fidelity of spiking responses to optical stimulation depends not only on the kinetics of the opsin protein, but also on expression patterns and strength, the biophysical characteristics of the cells being stimulated, and the network dynamics of the neural populations being stimulated^43,92^. Consequently, there may be considerable differences in optically-evoked responses between rodent and primate neural circuits. This underscores that New World primates are likely a closer functional approximation than rodents to the effect of optogenetic manipulation on rhesus, and perhaps human, neuronal firing patterns.

We note, however, that our recordings were conducted under isoflurane anesthesia which impacts cortical activity. In particular, isoflurane suppresses cortical firing rates, which prevented us from validating the hyperpolarizing opsins *in vivo*. Validation of these constructs will require future experiments which use a different anesthetic protocol which preserves spontaneous firing (e.g. using pentobarbital), which combine optogenetic excitation and inhibition in the same region, or in which photostimulation is performed in awake subjects. We anticipate that the precise evoked spiking activity would also differ as a function of many experimental parameters, including the viral titer used, the brain region injected, the level of opsin expression, the cell types being targeted and recorded from, and the parameters of photostimulation. Consequently, optical responses should be functionally validated via simultaneous electrophysiology in any particular experiment to validate the evoked responses.

### Long-term safety and efficacy of optogenetic constructs

Optogenetic manipulations of neural circuits show promise in helping to disentangle the pathology of psychiatric disorders, including depression, obsessive-compulsive disorder, anhedonia, anxiety, etc. (reviewed in^28^). However, an important step in this direction is establishing the long-term viability and safety of optogenetic constructs in a primate model. In four squirrel monkeys and three rhesus macaques, viral transfection of optogenetic constructs did not lead to any adverse health effects through at least one full year, as assessed using a combination of MR imaging, blood tests, and post-mortem histology. Furthermore, the injected opsins continued to express for at least one year in both squirrel monkeys and in rhesus macaques, and that optically-evoked spiking activity remained robust at least a full year with no significant reduction in efficacy over time.

### Motivation and outlook for optogenetics in squirrel monkeys and other primates

Within systems neuroscience, the rapid expansion of genetically-encoded, optogenetic tools has enabled targeted, functional manipulations across the phylogenetic tree^40,93–95^. These tools enable optical control of specific neuron types or circuit projections, enabling researchers to probe neural circuits in healthy and disease states^28^. Rodent models have so far benefited most abundantly; however, there is strong motivation to apply these tools in primates due to the richness and precision of primate behavior and to the translational relevance afforded by the homology between human and nonhuman primate brains^1,2^. For more complex, cognitively demanding behaviors beyond the repertoire of rodents or for translational questions where homology with the human brain is paramount, understanding neural circuits in primates is critical. The addition of optogenetic tools and viral targeting strategies to the primate toolkit will aid researchers in understanding the complex, neural computations which underlie perception, sophisticated cognition and decision making, and dextrous motor behavior across the primate lineage.

Squirrel monkeys are a long-established model system in biomedical research, utilized for decades to study learning and memory^96–98^, olfaction^99^, vision^100^, behavioral pharmacology^101,102^, addiction^103,104^, social behavior^105,106^, stress-induced physiological changes^107,108^, aging and protein aggregation^109^, Parkinson’s disease^110^, and other human diseases^111^. Their relatively large brains (Fig. 1a) display similar functional organization to rhesus monkeys and humans^112–114^, and researchers have leveraged this homology to study the neurophysiology of the vestibular system^115–117^, sleep^118^, audition^119,120^, vocalization^121–123^, motor control^124^, and many other neuroscientific questions.

We discuss four specific scientific opportunities afforded by the use of optogenetic tools within squirrel monkeys. First, within the particular context of motor control, we envision many long-standing lines of scientific inquiry where optogenetic tools would likely yield new insight. Squirrel monkeys possess a pseudo-opposable thumb for gripping objects and a central sulcus dividing functionally organized primary motor and somatosensory cortices. This biomechanical homology and similarities in functional organization facilitate straightforward comparison with homologous regions in rhesus macaques. Additionally, the somatosensory and motor systems in squirrel monkeys have been well mapped using microelectrode arrays, intrinsic signal imaging, and functional MRI^124–130^. Intracortical and subcortical connections among these areas have also been explored using retrograde and anterograde tracing techniques^125,129,131,132^. Optogenetic manipulations would facilitate functional dissection of these regions and their contributions to hand and limb movements. For example, spatially localized optogenetic inhibition could be used to instantaneously shutdown the contribution of Area 5, S1, M1, PMd, or PMv during the preparation and execution of reaching and grasping movements. Leveraging the anterograde transduction observed for the opsins we tested, injection in one region and optically exciting or suppressing downstream axonal terminals could be used to test the functional contributions of the many interconnections between these regions. For example, activating or suppressing the parietal reach region to M1 and PMd during reaching movements could elucidate the processes of state estimation and visual feedback that guide reaching movements. Similarly, optogenetic manipulation of cerebellar projections to thalamus, thalamocortical projections to M1, or reciprocal projections between 3a and M1 would inform models of how proprioceptive information is transformed by motor cortex during online feedback control^133,134^.

Second, changes to the functional organization have been characterized subsequent to injury due to local infarct^135–137^, cortical injury^138,139^, and dorsal column spinal cord lesions^140–143^. Nevertheless, characterization of this remapping is typically limited to passive observation of motor representations. Temporally precise and anatomically targeted causal manipulations would greatly enhance our functional understanding of the mechanisms of cortical remapping following injury.

Third, while squirrel monkeys are a long-established model in their own right within motor control and other neuroscientific domains, the scientific utility of optogenetic tools in squirrel monkeys also lies in providing causal tools for functional comparative neuroanatomy. Comparative efforts are greatly accelerated by concomitant developments in other primates, notably rhesus macaques and marmosets. Impressive recent progress has been made towards translating optogenetic tools to rhesus macaques (reviewed in^19,144,145^) and marmosets^7^. Macaques display close neuroanatomical homology with humans and are readily trained to perform a wide variety of cognitively sophisticated tasks. In macaques, some behavioral effects of stimulation have been surprisingly modest^6,12,13,19,84,86^, although notable effects have been reported, particularly when stimulating sensory areas^8–11^ and when specific circuit projections^14^ or cell types^15^ were targeted. This body of work underscores the need to develop refined targeting strategies to better understand the nuanced interaction between optogenetic manipulations and the endogenous circuit dynamics that leads to behavioral modification. Progress with viral tools for neural circuit targeting and manipulation has also recently been reported in the common marmoset, whose reproductive characteristics afford the creation of transgenic models that open a rich experimental landscape both in basic science and translational research^146,147^. As novel transgenic marmoset lines are developed^146,148,149^ and advanced tools for genome editing become widely available^150^, marmosets offer an attractive, complementary platform for combining genetic targeting, neuroanatomical mapping, awake-behaving neurophysiology, and sophisticated behavioral control^151–155^. Moreover, AAV can efficiently transfect neurons in marmosets^53^ and excitatory and inhibitory opsins readily express^7^.

To date, the vast majority of this comparative research has been histological (e.g.^129,156–161^), though additional techniques including electrophysiological recording, intracortical microstimulation, lesions, TMS, and functional MRI have been used to probe the differences among the nervous systems of New World and Old World primates and humans (e.g.^128,131,162–164^). By extending optogenetic tools to additional primate species, functional manipulations can be brought to bear on comparative analysis of neural organization among the primate lineages. It would be of particular interest to revisit the evolutionary differences in the corticospinal tract observed among New World and Old World primates. Moving from marmosets to squirrel monkeys to *Cebus* monkeys to macaques, one observes a progressively larger cortico-motorneuronal tract (comprised of motor cortical neurons that directly excite lower motor neurons in the ventral horn of the spinal cord), as well as a progressively greater hand dexterity and capacity for relatively independent finger movements^129,161,163–165^. However, the functional significance of different contributions to the corticospinal tract and other descending motor pathways remains unclear; this body comparative work is largely correlative in nature. A notable exception was recently reported by Kinoshita and colleagues^166^, who leveraged targeted, reversible expression of tetanus toxin to demonstrate that the phylogenetically “older” oligosynaptic pathway to motor neurons is still required for dextrous grasping in macaques. Targeted optogenetic manipulation of the descending motor pathways could definitively establish the relative functional contribution of descending pathways across primate species and their relationship to dextrous motor behaviors. Parallel efforts in multiple primate species will likely inform our understanding of neural structure and function in each species individually as well as within the larger evolutionary context.

Due to the considerable differences between rodents and humans, application of new tools for circuit neuroscience in primate models is essential to advancing our understanding of the human brain and of brain disorders^1^. This study highlights the potential for precise optical manipulation of specific circuits in squirrel monkeys, a well-established model in biomedical science, featuring close anatomical, behavioral, and genetic homology with macaques and humans. While the optimal viruses, promoters, proteins, and targeting strategies employed will likely vary with context, novel elements of the toolkit for optogenetic manipulation developed here for squirrel monkeys will likely translate to other New World primates and to rhesus macaques, enabling similar strategies for projection targeting and high-fidelity optical control. In parallel with rhesus macaques, New World primate models can facilitate the translation of an ever-growing collection of precision tools available for circuit manipulation and interrogation^95^, presenting an exceptional opportunity to dissect neural circuit function and disease.

## Methods In Brief

All procedures followed approved Stanford University IACUC protocols. Optogenetic protocols are described online at the Optogenetics Resource Center, http://www.stanford.edu/group/dlab/optogenetics/. **Injections**: Twelve adult female squirrel monkeys (*Saimiri sciureus*) were anesthetized with ketamine and isoflurane and then relocated to an operating room where sterile surgical practices were followed. Opsin constructs were injected stereotactically using a microsyringe pump system. Three rhesus macaques were injected using a standard electrophysiology microdrive and custom injection system while awake in a primate chair. **Recording**: After at least six weeks post-injection, 4 squirrel monkeys were re-anesthetized for intraoperative optical stimulation and electrophysiological recording using hand-built fiber optrodes connected to a portable multichannel recording and laser stimulation cart. Anesthetized recording procedures were followed immediately by perfusion. Rhesus macaques were stimulated via a custom coaxial recoding/stimulation optrode^167^ while awake in a primate neurophysiology rig. **Perfusion and Histology**: All animals were deeply anesthetized with ketamine and isoflurane, euthanized with Beuthanasia, and then perfused with heparinized ice-cold PBS and 4% paraformaldehyde. Histological sections were prepared in standard fashion, stained, and imaged using confocal laser microscopy.

## Methods

All experiments were approved by the Stanford University Institutional Animal Care and Use Committee and the Stanford University Administrative Panel on Biosafety.

### Virus production

Standard protocols were used to produce viral vectors and viruses (see http://optogenetics.org). AAVs (1–4 * 10^12^ genome copies per mL) were packaged by the University of North Carolina viral vector core. All viruses had been tested in rodents and expression was assessed by standard histological measurements before injecting them into monkeys. The majority of our injections used AAV2/5, which refers to a pseudotyped AAV that combines the genome of AAV2 with the capsid of AAV5 to improve transduction efficiency and neural tropism (https://www.addgene.org/viral-vectors/aav/aav-guide/). By convention, the pseudotyped AAV is referred to by its capsid component (i.e. AAV2/2 is referred to as AAV2, AAV 2/5 is referred to as AAV5 and AAV2/8 is referred to as AAV8) and is assumed to have the same tropism/spread within brain tissue. For WGA-Cre projection targeting constructs, we employed AAV2/2 (AAV2) and AAV2/8 (AAV8) to combine two different serotypes within a single brain, with the EF1*α* promoter responsible for driving strong expression of Cre (and therefore AAV2 was persumed drive sufficient expression). One injection was also performed with LT-HSV^47^.

### Virus injections and targeting

#### Squirrel monkeys

Twelve adult female squirrel monkeys (*Saimiri sciureus sciureus*) between 10–18 years old (mean 13.6 ± 2.6 std. dev. years) were sedated with ketamine and 0.5–4% isoflurane. One or more relatively large rectangular craniotomies were performed over stereotactically-defined injection sites under sterile conditions. A stereotactic positioner (Kopf Instruments) was used to advance a 100-*μ*l syringe (1710TLL; Hamilton) and a 32-gauge injection needle (Hamilton Company) to a depth of 3 mm beyond dura. Injections of 1 *μ*l were made every 1 mm at a rate of 100 nl/min, controlled by a programmable syringe pump (World Precision Instruments). At least 6 weeks post-injection were allowed to enable opsin expression, based on rodent and rhesus expression times^6^. We housed the squirrel monkeys for a few days post-surgery in an adjacent but separate cage and then returned them to their group home cages.

We used interaural stereotactic coordinates to target each injection to one of primary somatosensory cortex (S1), primary motor cortex (M1), dorsal premotor cortex (PMd), or parietal cortex. The large craniotomies exposed the relatively translucent dura and facilitated visualization of the central sulcus and nearby sulcal fiducials. S1 and M1 were targeted posterior and anterior to the central sulcus respectively. The parietal injection was targeted near the parietal sulcus and the PMd injections were targeted relative to the central and arcuate sulci and the precentral dimple. The injection locations were also validated histologically. We validated that S1 and M1 injections were located on the postcentral and precentral gyri and that parietal cortex was located near the parietal sulcus. PMd was validated to lie 11–15 mm anterior to the central sulcus but posterior to the arcuate sulcus. We were not able to functionally validate PMd using intracortical microstimulation (i.e. higher thresholds to evoke movement than in M1) or electrophysiologically (e.g. higher proportion of neurons displaying robust preparatory activity in instructed delay reaching tasks). However, we did note a reduction in the proportion of large neurons in layer 5 (likely Betz cells) in the targeted PMd relative to M1^157^. Examples of M1 Betz cells are shown in Figs 1e and 4a.

#### Rhesus macaques

Three adult rhesus macaque monkeys (*Macaca mulatta*), one female (rhesus monkey D) and two males (rhesus monkeys Q and O), ranging in age from 6–15 years, were implanted under isoflurane anesthesia with a recording cylinder perpendicular to the skull over primary motor and premotor cortex. In monkeys D and O, multiple small craniotomies were made within the cylinder; in monkey Q, a full craniotomy was performed. In all cases, the dura was left intact. During the following days, we injected virus using an adapted microinfusion system^168^ as described in^6^. A hydraulic micromanipulator (Narishige Group) lowered the needles through a guide tube attached to a Crist grid and injections of 1 *μ*l were made every 1 mm. After injecting at each depth, we waited 10 min before moving deeper. Following completion of the deepest injection (6–10 mm depth), the micromanipulator withdrew the injection needle at a speed of 1–4 mm/min. Injections were made in up to 14 locations within each monkey’s cylinder with at least 1–2 mm of separation between adjacent sites.

We used interaural stereotactic coordinates to target each injection to M1 and/or PMd (Supp. Table 1). We derived these coordinates via consultation with histological atlases and with previously validated coordinates used in prior surgeries. We extensively validated these regions electrophysiologically during reaching tasks. Both M1 and PMd exhibited modulation during reaching over time and across reach directions. Both areas were reliably modulated by palpation of the contralateral upper arm and shoulder. PMd exhibited more preparatory activity in advance of movement. Using intracortical microstimulation (333 Hz biphasic pulses, c.f.^169^), M1 exhibited markedly lower thresholds (10 uA to 50 uA) to evoke movement than PMd (70 uA to 180 uA), but eye movements were not evoked.

### Optical stimulation and neural recordings

#### Squirrel monkeys

Four squirrel monkeys were sedated with ketamine and anesthetized with isoflurane and placed in a stereotax. Craniotomies were reopened under sterile conditions over the previous injection sites. We constructed optrodes which were 200 *μ*m flat-cut optical fibers (Thorlabs) glued to high-impedance (3–7 MΩ) tungsten electrodes (Serial number UEWLGCSEEN1E, Frederick Haer) with the electrode tip leading the fiber by 100–400 *μ*m). Optrodes were advanced using a stereotactic positioner (David Kopf Instruments). Optical stimulation and electrophysiology were performed using a custom-built mobile electrophysiology rig built on a rolling cart (Bretford Manufacturing). See Supp. Fig. 4 for component schematic. Optical stimulation was computer controlled using a Simulink real-time xPC target system, which triggered laser stimulation pulses via a digital-to-analog card (National Instruments) that controlled a blue (473 nm, Sanctity, SVL-473-0100), green (561 nm, CrystaLasers, CL-2000), or red (534 nm, CrystaLasers, DL-635) laser in analog mode. Electrode signals were recorded using the Cerebus system (Blackrock Microsystems).

We advanced the electrode while pulsing the stimulating laser for 2 ms at a rate of 1 Hz. This facilitated the detection of optogenetically-evoked spiking activity at the electrode. While light-responsive single- and multi-units were readily recorded at the electrode, this inherently biased sample of neuronal responses precludes quantification of the fraction of light-responsive units in the region near the injection site. For computing evoked spike counts during pulsed stimulation, as in Fig. 5c,d, a spike was counted as being evoked by a particular laser pulse if it occurred in a window beginning at laser onset and extending 7.5 ms forwards in time. This window was selected to be nearly as large as possible while avoiding overlap for successive pulses with 130 Hz stimulation so that the same evoked spiking window could be employed for all stimulation frequencies.

For extended stimulation with pulse trains, 50 Hz trains of 2 ms pulses were delivered for 5 sec spaced with 10 sec pauses, for a total of 360 repetitions over the 90-minute experiment, or 60 repetitions per 15-minute epoch. For extended stimulation with continuous pulses, 200 ms pulses were delivered spaced with 800 ms pauses, for a total of 5400 repetitions over the 90-minute experiment, or 900 repetitions in each 15-minute epoch. Laser power was fixed at 3 mW (95.5 mW/mm^2^) for the duration of both experiments. Electrophysiological responses were quantified using custom MATLAB analysis code. Spike trains were smoothed with either a 10 ms Gaussian filter (for frequency modulation) or a 20 ms Gaussian filter and then averaged over trials. Spike counts during stimulation were compared to pre-stimulation time periods using the non-parametric Wilcoxon signed-rank test for paired samples. Changes in spike counts evoked by continuous vs. pulsed stimulation were compared using the non-parametric rank-sum test for unpaired samples.

Frequency modulation stimulation patterns were generated as follows: first, the pulse rate as a function of time was specified to jump from 0 Hz to 80 Hz (the center frequency) and persisted at 80 Hz for 1 second to allow onset transients to decay. After 1 second, frequency modulation varied the pulse rate sinusoidally between 30 and 130 Hz at a signal frequency determined by the condition, ranging from 1–14 Hz. After 3 seconds of frequency modulation, another 1 second of 80 Hz constant pulse rate followed, and then the pulse rate returned to 0. We then used this pulse rate timeseries as the average rate of a highly regular gamma process of order 40 from which we generated the pulse times on each trial.

#### Rhesus macaques

Optogenetic stimulation in rhesus monkeys Q and O differed in that all stimulation was done with awake monkeys comfortably seated in a primate chair within a dim, quiet neurophysiology rig. We utilized either hand-built optrodes (rhesus monkey Q) as described above or a custom coaxial optrode designed to minimize cortical damage^167^ (rhesus monkeys Q and O). The optrode was positioned over the injection location and lowered into cortex until background neural activity and optically-evoked responses from the local population could be detected in the voltage recordings. Stimulation occurred in the inter-trial intervals after a random subset of trials of a reaching task performed in the course of other experiments, during which time the monkeys were waiting passively for the next trial to begin. Stimulation was delivered at 3 mW (95.5 mW/mm^2^) as single 200 ms continuous pulses at 561 nm (CrystaLasers, CL-2000). We measured the average firing rate of single or multiunit activity recorded on the optrode by counting spikes in the 200 ms stimulation window and over repeated stimulated and non-stimulated trials. We computed the difference in firing rate between stimulated and non-stimulated trials and computed 95% confidence intervals from the Student’s T distribution purely for display purposes. To test whether the size of optically-evoked changes in firing rates was changing over time, we performed a least-squares linear regression fit to the change in firing rate vs. days from injection. The 95% confidence intervals on the coefficients were generated from the F-distribution.

### Both squirrel monkeys and rhesus macaques

Electrode voltages were unity-gain buffered at a head stage near the recording electrode and then filtered using the Cerebus “spike medium” filter (250 Hz 4-pole high-pass filter). We ensured monitored noise levels on the electrode and ensured that the RMS noise voltage was below 10 µV at all times, using appropriate grounding, referencing, and EMI shielding techniques. High-pass filtered spike signals were then thresholded online using a threshold which was set manually to isolate the waveforms of an individual neuron (single unit) or collection of several neurons (multi-unit) from the noise. This threshold was always at least> 4x RMS or <−4x RMS to ignore background noise or poorly isolated neural “hash”. We did not include data from any single or multi-unit waveforms which were not reliably isolated from the noise for the duration of the procedure. In some cases, the isolated spiking waveforms were separated into multiple individual single units using time-amplitude windows (Plexon Offline Sorter). The single unit and multi-unit examples included were selected on the basis of optical responsiveness. Stimulation paradigms were designed using MATLAB (Mathworks). Laser power at fiber tip was set to 0.5–5 mW as measured with a power meter (Thorlabs). Pulse trains ranged in frequency from 20 Hz to 130 Hz and used 2 ms pulses. Continuous pulses lasted for 200–400 ms.

When stimulating in ChR2(H134R)-transfected regions, we also employed red-wavelength control stimulation to verify that recorded responses were due to optogenetic modulation and not artifactual. In only these control experiments, we set the threshold for spike detection liberally at 3x RMS voltage (24 μV). This threshold was closer to the electrical noise and much lower than what was used to isolate spiking neurons for non-control experiments. By recording the local neural “hash”, this enabled detection of minute changes in local neural activity, increasing the sensitivity of the control experiment to non-specific modulation by the laser itself. We compared the time-averaged firing rate during stimulation to each trial’s pre-stimulation baseline using a paired two-sided sign-rank test.

### Structural MR Imaging

At least one year after virus injection, structural brain images from two squirrel monkeys were acquired on a General Electric Signa 3.0 Tesla system with protocols developed specifically for squirrel monkeys as described previously^170^. The monkeys were scanned under anesthesia induced by subcutaneous injection of 20 mg/kg ketamine hydrochloride, 4 mg/kg xylazine hydrochloride, and 0.04 mg/kg atropine sulfate. Body temperature was monitored and maintained within the normal range using a cushioned heat pad. Earplugs protected against scanner-generated noise. Three types of imaging sequences were acquired per monkey: a T1-weighted 3D IR-FSPGR (TR/TE/TI 8.3/2.8/500 ms), a T2-weighted FAST SPIN ECHO sequence (T2 FSE) (TR/TE 3200/206.6 ms) and a T2-FLAIR sequence (TR/TE 6000/180.9 ms). All scans were acquired in the coronal plane, with field of view 8 cm, matrix 256 × 256, voxel size 0.31 × 0.31 × 0.5 mm. Images were digitally resliced in the sagittal plane for presentation. Inversion recovery prepared protocols, such as the 3D T1-SPGR sequence acquired in this study, have been previously shown to maximize gray versus white matter contrast^171^.

### Histology and imaging

At least 6 weeks post-injection, squirrel monkeys and rhesus macaques were deeply anesthetized with ketamine and isoflurane, euthanized with Beuthanasia, then perfused with heparinized ice-cold PBS followed by ice-cold 4% paraformaldehyde. Extracted brains and spinal cords were fixed overnight in 4% paraformaldehyde and transferred to 30% sucrose solution for 48–72 hours at 4 °C. Brains and spinal cord were sectioned into blocks and frozen. 40 *μ*m thick sections were then cut on a freezing microtome (Leica SM2000R) and preserved in cryoprotectant (25% glycerol and 30% ethylene glycol in PBS) at 4 °C. Sections were processed using standard immunohistochemical procedures previously reported in^6,172^. In brief, sections were incubated overnight at 4 °C with the primary antibodies rabbit anti-CaMKII*α* 1:50 (Santa Cruz), mouse anti-NeuN 1:500 (Millipore) and/or rabbit anti-GFAP 1:500 (Millipore), in PBS with 0.01% Triton X-100 (wt/vol) and 3% normal donkey serum (wt/vol). Sections were then washed in PBS and incubated in secondary antibodies (1:1000) conjugated to Cy3, Cy5, Alexa 647, Alexa 594, or DAPI (Jackson Immuno Research) for three hours at room temperature in PBS with 0.01% Triton X-100 (wt/vol) and 3% normal donkey serum (wt/vol). Sections were washed in PBS and incubated in DAPI (1:50,000) for 30 mins, then washed again and mounted on slides with PVA-DABCO. Fluorescence images were acquired using a Leica TCS SP5 confocal scanning laser microscope with 10x air and 40x/1.4 NA oil objectives. Large field coronal images were collected using either a 10x, 20x, or 40x objective, tiling stage control, and stitched together in software (Fiji). For each slice, we stained for DAPI and/or NeuN and/or GFAP to address specific questions about neural tropism and toxicity of the viral serotype/promoter/opsin used.

In the vicinity of the injection site, the coronal slice with the brightest fluorescence was selected to demonstrate the expression. In a few cases, the same opsin/promoter combination was injected into more than one squirrel monkey. We included images of the first squirrel monkey for whom histology was performed. In rare cases where difficulties with perfusion, slicing, or staining negatively impacted the integrity of the brain slices or the quality of the fluorescent stain, we selected an alternative slice, possibly from another squirrel monkey injected with the same construct.

### Data availability

The datasets generated during and/or analyzed during the current study are available from the corresponding author on reasonable request.

## Electronic supplementary material

Supplementary Materials

## References

[1] Belmonte Juan Carlos Izpisua, Callaway Edward M., Caddick Sarah J., Churchland Patricia, Feng Guoping, Homanics Gregg E., Lee Kuo-Fen, Leopold David A., Miller Cory T., Mitchell Jude F., Mitalipov Shoukhrat, Moutri Alysson R., Movshon J. Anthony, Okano Hideyuki, Reynolds John H., Ringach Dario L., Sejnowski Terrence J., Silva Afonso C., Strick Peter L., Wu Jun, Zhang Feng (2015). Brains, Genes, and Primates. Neuron.

[2] Huang, L., Merson, T. D. & Bourne, J. A. *In vivo* whole brain, cellular and molecular imaging in nonhuman primate models of neuropathology, en. *Neuroscience and biobehavioral reviews* **66**, 104118, ISSN: 0149–7634, 1873–7528 (2016). 10.1016/j.neubiorev.2016.04.009 27151822

[3] Kaas Jon H. (2006). Evolution of the neocortex. Current Biology.

[4] Chaplin, T. A., Yu, H.-H., Soares, J. G. M., Gattass, R. & Rosa, M. G. P. A conserved pattern of differential expansion of cortical areas in simian primates, en. *The Journal of neuroscience*: *the official journal of the Society for Neuroscience* **33**, 15120–15125, ISSN: 0270–6474, 1529–2401 (2013). 10.1523/JNEUROSCI.2909-13.2013 PMC6618405 24048842

[5] Han Xue, Qian Xiaofeng, Bernstein Jacob G., Zhou Hui-hui, Franzesi Giovanni Talei, Stern Patrick, Bronson Roderick T., Graybiel Ann M., Desimone Robert, Boyden Edward S. (2009). Millisecond-Timescale Optical Control of Neural Dynamics in the Nonhuman Primate Brain. Neuron.

[6] Diester Ilka, Kaufman Matthew T, Mogri Murtaza, Pashaie Ramin, Goo Werapong, Yizhar Ofer, Ramakrishnan Charu, Deisseroth Karl, Shenoy Krishna V (2011). An optogenetic toolbox designed for primates. Nature Neuroscience.

[7] MacDougall, M. *et al*. Optogenetic manipulation of neural circuits in awake marmosets, en. *Journal of neurophysiology*, jn.00197.2016, ISSN: 0022–3077, 1522–1598 (2016). 10.1152/jn.00197.2016 PMC5023415 27334951

[8] Jazayeri, M., Lindbloom-Brown, Z. & Horwitz, G. D. Saccadic eye movements evoked by optogenetic activation of primate VI. *Nature neuroscience* **15**, 1368–1370, ISSN: 1097–6256, 1546–1726 (2012). 10.1038/nn.3210 PMC3458167 22941109

[9] May, T. *et al*. Detection of optogenetic stimulation in somatosensory cortex by non-human primates-towards artificial tactile sensation, en. *PloS one* **9**, e114529, ISSN: 1932–6203 (2014). 10.1371/journal.pone.0114529 PMC4277269 25541938

[10] Afraz, A., Boyden, E. S. & DiCarlo, J. J. Optogenetic and pharmacological suppression of spatial clusters of face neurons reveal their causal role in face gender discrimination, en. *Proceedings of the National Academy of Sciences of the United States of America* **112**, 6730–*6735*, ISSN: 0027–8424, 1091–6490 (2015). 10.1073/pnas.1423328112 PMC4450412 25953336

[11] Dai, J., Brooks, D. I. & Sheinberg, D. L. Optogenetic and Electrical Microstimulation Systematically Bias Visuospatial Choice in Primates. *Current biology*: *CB* **24**, 63–69, ISSN: 0960–9822, 1879–0445 (2013). 10.1016/j.cub.2013.11.011 24332543

[12] Galvan, A., Hu, X., Smith, Y. & Wichmann, T. Effects of Optogenetic Activation of Corticothalamic Terminals in the Motor Thalamus of Awake Monkeys, en. *The Journal of neuroscience*: *the official journal of the Society for Neuroscience* **36**, 3519–3530, ISSN: 0270–6474, 1529–2401 (2016). 10.1523/JNEUROSCI.4363-15.2016 PMC4804009 27013680

[13] Lu, Y. *et al*. Optogenetically-Induced Spatiotemporal Gamma Oscillations and Neuronal Spiking Activity in Primate Motor Cortex, en. *Journal of neurophysiology*, jn.00792.2014, ISSN: 0022–3077, 1522–1598 (2015). 10.1152/jn.00792.2014 PMC4461886 25761956

[14] Inoue, K.-L, Takada, M. & Matsumoto, M. Neuronal and behavioural modulations by pathway-selective optogenetic stimulation of the primate oculomotor system. *Nature communications* **6**, 1–7 (2015). 10.1038/ncomms9378 PMC4595751 26387804

[15] Stauffer William R., Lak Armin, Yang Aimei, Borel Melodie, Paulsen Ole, Boyden Edward S., Schultz Wolfram (2016). Dopamine Neuron-Specific Optogenetic Stimulation in Rhesus Macaques. Cell.

[16] El-Shamayleh, Y., Ni, A. M. & Horwitz, G. D. Strategies for targeting primate neural circuits with viral vectors, en. *Journal of neurophysiology* **116**, 122–134, ISSN: 0022–3077, 1522–1598 (2016). 10.1152/jn.00087.2016 PMC4961743 27052579

[17] Nassi, J. J., Cepko, C. L., Born, R. T. & Beier, K. T. Neuroanatomy goes viral! en. *Frontiers in neuroanatomy* **9**, 80, ISSN: 1662–5129 (2015). 10.3389/fnana.2015.00080 PMC4486834 26190977

[18] Dai Ji, Ozden Ilker, Brooks Daniel I., Wagner Fabien, May Travis, Agha Naubahar S., Brush Benjamin, Borton David, Nurmikko Arto V., Sheinberg David L. (2015). Modified toolbox for optogenetics in the nonhuman primate. Neurophotonics.

[19] Gerits, A. & Vanduffel, W. Optogenetics in primates: a shining future? *Trends in genetics*: *TIG* **29**, 403–411, ISSN: 0168–9525 (2013). 10.1016/j.tig.2013.03.004 23623742

[20] Abee C. R. (2000). Squirrel Monkey (Saimiri spp.) Research and Resources. ILAR Journal.

[21] Gillis, T. E., Janes, A. C. & Kaufman, M. J. Positive reinforcement training in squirrel monkeys using clicker training, en. *American journal of primatology* **74**, 712–720, ISSN: 0275–2565, 1098–2345 (2012). 10.1002/ajp.22015 PMC3412074 22553135

[22] Williams, L. & Glasgow, M. Squirrel monkey behavior in research. *ILAR journal/National Research Council*, *Institute of Laboratory Animal Resources* **41**, 26–36, ISSN: 1084–2020 (2000). 10.1093/ilar.41.1.26 11421221

[23] Abee C. R. (1989). The Squirrel Monkey in Biomedical Research. ILAR Journal.

[24] Candland, D. K. *et al*. Social Structure of the Squirrel Monkey (Samiri sciureus, iquitos): Relationships among Behavior, Heartrate, and Physical Distance. *Folia primatologica; international journal of primatology* **20**, 211–240, ISSN: 0015–5713 (1973). 10.1159/000155576 4207776

[25] Andrews Michael W. (1993). Video-Task Paradigm Extended toSaimiri. Perceptual and Motor Skills.

[26] Rajasethupathy, P., Ferenczi, E. & Deisseroth, K. Targeting Neural Circuits, en. *Cell* **165**, 524–534, ISSN: 0092–8674, 1097–4172 (2016). 10.1016/j.cell.2016.03.047 PMC5296409 27104976

[27] Lerner, T. N., Ye, L. & Deisseroth, K. Communication in Neural Circuits: Tools, Opportunities, and Challenges, en. *Cell* **164**, 1136–1150, ISSN: 0092–8674, 1097–4172 (2016). 10.1016/j.cell.2016.02.027 PMC5725393 26967281

[28] Deisseroth Karl (2014). Circuit dynamics of adaptive and maladaptive behaviour. Nature.

[29] Tenenbaum, L., Lehtonen, E. & Monahan, P. E. Evaluation of risks related to the use of adeno-associated virus-based vectors. *Current gene therapy* **3**, 545–565, ISSN: 1566–5232 (2003). 10.2174/1566523034578131 14683451

[30] Mezzina, M. & Merten, O.-W. Adeno-associated viruses. *Methods in molecular biology* **737**, 211–234, ISSN: 1064–3745, 1940–6029 (2011). 10.1007/978-1-61779-095-9_9 21590399

[31] Li, H. *et al*. Assessing the potential for AAV vector genotoxicity in a murine model. *Blood* **117**, 3311–3319, ISSN: 0006–4971, 1528–0020 (2011). 10.1182/blood-2010-08-302729 PMC3069673 21106988

[32] Romano, G. Development of safer gene delivery systems to minimize the risk of insertional mutagenesis-related malignancies: a critical issue for the field of gene therapy. *ISRN oncology* **2012**, 616310, ISSN: 2090–5661, 2090–567X (2012). 10.5402/2012/616310 PMC3512301 23209944

[33] Wu, Z., Asokan, A. & Samulski, R. J. Adeno-associated virus serotypes: vector toolkit for human gene therapy. *Molecular therapy*: *the journal of the American Society of Gene Therapy* **14**, 316–327, ISSN: 1525–0016 (2006). 10.1016/j.ymthe.2006.05.009 16824801

[34] High, K. A. & Aubourg, P. rAAV human trial experience. *Methods in molecular biology* **807**, 429–457, ISSN: 1064–3745, 1940–6029 (2011). 10.1007/978-1-61779-370-7_18 22034041

[35] Taymans Jean-Marc, Vandenberghe Luk H., Haute Chris Van Den, Thiry Irina, Deroose Christophe M., Mortelmans Luc, Wilson James M., Debyser Zeger, Baekelandt Veerle (2007). Comparative Analysis of Adeno-Associated Viral Vector Serotypes 1, 2, 5, 7, And 8 in Mouse Brain. Human Gene Therapy.

[36] Aschauer, D. F., Kreuz, S. & Rumpel, S. Analysis of transduction efficiency, tropism and axonal transport of AAV serotypes 1, 2, 5, 6, 8 and 9 in the mouse brain. *PloS one* **8**, e76310, ISSN: 1932–6203 (2013). 10.1371/journal.pone.0076310 PMC3785459 24086725

[37] Cearley Cassia N, Vandenberghe Luk H, Parente Michael K, Carnish Erin R, Wilson James M, Wolfe John H (2008). Expanded Repertoire of AAV Vector Serotypes Mediate Unique Patterns of Transduction in Mouse Brain. Molecular Therapy.

[38] Markakis Eleni A, Vives Kenneth P, Bober Jeremy, Leichtle Stefan, Leranth Csaba, Beecham Jeff, Elsworth John D, Roth Robert H, Samulski R Jude, Redmond D Eugene (2010). Comparative Transduction Efficiency of AAV Vector Serotypes 1–6 in the Substantia Nigra and Striatum of the Primate Brain. Molecular Therapy.

[39] Boyden, E. S., Zhang, F., Bamberg, E., Nagel, G. & Deisseroth, K. Millisecond-timescale, genetically targeted optical control of neural activity. *Nature neuroscience* **8**, 1263–1268, ISSN: 1097–6256 (2005). 10.1038/nn1525 16116447

[40] Yizhar, O., Fenno, L. E., Davidson, T. J., Mogri, M. & Deisseroth, K. Optogenetics in neural systems, en. *Neuron* **71**, 9–34, ISSN: 0896–6273, 1097–4199 (2011). 10.1016/j.neuron.2011.06.004 21745635

[41] Gradinaru Viviana, Zhang Feng, Ramakrishnan Charu, Mattis Joanna, Prakash Rohit, Diester Ilka, Goshen Inbal, Thompson Kimberly R., Deisseroth Karl (2010). Molecular and Cellular Approaches for Diversifying and Extending Optogenetics. Cell.

[42] Chow Brian Y., Han Xue, Dobry Allison S., Qian Xiaofeng, Chuong Amy S., Li Mingjie, Henninger Michael A., Belfort Gabriel M., Lin Yingxi, Monahan Patrick E., Boyden Edward S. (2010). High-performance genetically targetable optical neural silencing by light-driven proton pumps. Nature.

[43] Mattis Joanna, Tye Kay M, Ferenczi Emily A, Ramakrishnan Charu, O'Shea Daniel J, Prakash Rohit, Gunaydin Lisa A, Hyun Minsuk, Fenno Lief E, Gradinaru Viviana, Yizhar Ofer, Deisseroth Karl (2011). Principles for applying optogenetic tools derived from direct comparative analysis of microbial opsins. Nature Methods.

[44] Ting, J. T. & Feng, G. Development of transgenic animals for optogenetic manipulation of mammalian nervous system function: progress and prospects for behavioral neuroscience. *Behavioural brain research* **255**, 3–18, ISSN: 0166–4328, 1872–7549 (2013). 10.1016/j.bbr.2013.02.037 PMC4030747 23473879

[45] Witten Ilana B., Steinberg Elizabeth E., Lee Soo Yeun, Davidson Thomas J., Zalocusky Kelly A., Brodsky Matthew, Yizhar Ofer, Cho Saemi L., Gong Shiaoching, Ramakrishnan Charu, Stuber Garret D., Tye Kay M., Janak Patricia H., Deisseroth Karl (2011). Recombinase-Driver Rat Lines: Tools, Techniques, and Optogenetic Application to Dopamine-Mediated Reinforcement. Neuron.

[46] Chamberlin, N. L., Du, B., de Lacalle, S. & Saper, C. B. Recombinant adeno-associated virus vector: use for transgene expression and anterograde tract tracing in the CNS. *Brain research* **793**, 169–175, ISSN: 0006–8993 (1998). 10.1016/s0006-8993(98)00169-3 PMC4961038 9630611

[47] Berges, B. K., Wolfe, J. H. & Fraser, N. W. Transduction of brain by herpes simplex virus vectors. *Molecular therapy*: *the journal of the American Society of Gene Therapy* **15**, 20–29, ISSN: 1525–0016 (2007). 10.1038/sj.mt.6300018 17164771

[48] Yizhar Ofer, Fenno Lief E., Prigge Matthias, Schneider Franziska, Davidson Thomas J., O’Shea Daniel J., Sohal Vikaas S., Goshen Inbal, Finkelstein Joel, Paz Jeanne T., Stehfest Katja, Fudim Roman, Ramakrishnan Charu, Huguenard John R., Hegemann Peter, Deisseroth Karl (2011). Neocortical excitation/inhibition balance in information processing and social dysfunction. Nature.

[49] Sohal, V. S., Zhang, F., Yizhar, O. & Deisseroth, K. Parvalbumin neurons and gamma rhythms enhance cortical circuit performance, en. *Nature* **459**, 698–702, ISSN: 0028–0836, 1476–4687 (2009). 10.1038/nature07991 PMC3969859 19396159

[50] Tye Kay M., Prakash Rohit, Kim Sung-Yon, Fenno Lief E., Grosenick Logan, Zarabi Hosniya, Thompson Kimberly R., Gradinaru Viviana, Ramakrishnan Charu, Deisseroth Karl (2011). Amygdala circuitry mediating reversible and bidirectional control of anxiety. Nature.

[51] Azim E, Jiang J, Alstermark B, Jessell TM (2014). Skilled reaching relies on a V2a propriospinal internal copy circuit, en. Nature.

[52] Rajasethupathy Priyamvada, Sankaran Sethuraman, Marshel James H., Kim Christina K., Ferenczi Emily, Lee Soo Yeun, Berndt Andre, Ramakrishnan Charu, Jaffe Anna, Lo Maisie, Liston Conor, Deisseroth Karl (2015). Projections from neocortex mediate top-down control of memory retrieval. Nature.

[53] Watakabe Akiya, Ohtsuka Masanari, Kinoshita Masaharu, Takaji Masafumi, Isa Kaoru, Mizukami Hiroaki, Ozawa Keiya, Isa Tadashi, Yamamori Tetsuo (2015). Comparative analyses of adeno-associated viral vector serotypes 1, 2, 5, 8 and 9 in marmoset, mouse and macaque cerebral cortex. Neuroscience Research.

[54] Stepniewska, I., Preuss, T. M. & Kaas, J. H. Thalamic connections of the primary motor cortex (Ml) of owl monkeys, en. *The Journal of comparative neurology* **349**, 558–582, ISSN: 0021–9967 (1994). 10.1002/cne.903490405 7532193

[55] Salegio E A, Samaranch L, Kells A P, Mittermeyer G, San Sebastian W, Zhou S, Beyer J, Forsayeth J, Bankiewicz K S (2012). Axonal transport of adeno-associated viral vectors is serotype-dependent. Gene Therapy.

[56] Ciesielska Agnieszka, Mittermeyer Gabriele, Hadaczek Piotr, Kells Adrian P, Forsayeth John, Bankiewicz Krystof S (2011). Anterograde Axonal Transport of AAV2-GDNF in Rat Basal Ganglia. Molecular Therapy.

[57] Eberling Jamie L., Kells Adrian P., Pivirotto Philip, Beyer Janine, Bringas John, Federoff Howard J., Forsayeth John, Bankiewicz Krystof S. (2009). Functional Effects of AAV2-GDNF on the Dopaminergic Nigrostriatal Pathway in Parkinsonian Rhesus Monkeys. Human Gene Therapy.

[58] Kells A. P., Hadaczek P., Yin D., Bringas J., Varenika V., Forsayeth J., Bankiewicz K. S. (2009). Efficient gene therapy-based method for the delivery of therapeutics to primate cortex. Proceedings of the National Academy of Sciences.

[59] Castle, M. J., Gershenson, Z. T., Giles, A. R., Holzbaur, E. L. F. & Wolfe, J. H. Adeno-associated virus serotypes 1, 8, and 9 share conserved mechanisms for anterograde and retrograde axonal transport, en. *Human gene therapy* **25**, 705–720, ISSN: 1043–0342, 1557–7422 (2014). 10.1089/hum.2013.189 PMC4137353 24694006

[60] Kaspar, B. K., Lladó, J., Sherkat, N., Rothstein, J. D. & Gage, F. H. Retrograde viral delivery of IGF-1 prolongs survival in a mouse ALS model, en. *Science* **301**, 839–842, ISSN: 0036–8075, 1095–9203 (2003). 10.1126/science.1086137 12907804

[61] Passini Marco A., Macauley Shannon L., Huff Michael R., Taksir Tatyana V., Bu Jie, Wu I-Huan, Piepenhagen Peter A., Dodge James C., Shihabuddin Lamya S., O'Riordan Catherine R., Schuchman Edward H., Stewart Gregory R. (2005). AAV Vector-Mediated Correction of Brain Pathology in a Mouse Model of Niemann–Pick A Disease. Molecular Therapy.

[62] Rothermel, M. & Brunert, D. Transgene Expression in Target-Defined Neuron Populations Mediated by Retrograde Infection with Adeno-Associated Viral Vectors. *The Journal of …* **33**, 15195–15206 (2013). 10.1523/JNEUROSCI.1618-13.2013 PMC3776063 24048849

[63] Burger Corinna, Gorbatyuk Oleg S., Velardo Margaret J., Peden Carmen S., Williams Philip, Zolotukhin Sergei, Reier Paul J., Mandel Ronald J., Muzyczka Nicholas (2004). Recombinant AAV Viral Vectors Pseudotyped with Viral Capsids from Serotypes 1, 2, and 5 Display Differential Efficiency and Cell Tropism after Delivery to Different Regions of the Central Nervous System. Molecular Therapy.

[64] Yasuda T., Miyachi S., Kitagawa R., Wada K., Nihira T., Ren Y.-R., Hirai Y., Ageyama N., Terao K., Shimada T., Takada M., Mizuno Y., Mochizuki H. (2007). Neuronal specificity of α-synuclein toxicity and effect of Parkin co-expression in primates. Neuroscience.

[65] Zhang Siyu, Xu Min, Chang Wei-Cheng, Ma Chenyan, Hoang Do Johnny Phong, Jeong Daniel, Lei Tiffany, Fan Jiang Lan, Dan Yang (2016). Organization of long-range inputs and outputs of frontal cortex for top-down control. Nature Neuroscience.

[66] Towne, C., Schneider, B. L., Kieran, D., Redmond Jr., D. E. & Aebischer, P. Efficient transduction of non-human primate motor neurons after intramuscular delivery of recombinant AAV serotype 6, en. *Gene therapy* **17**, 141–146, ISSN: 0969–7128, 1476–5462 (2010). 10.1038/gt.2009.119 19727139

[67] San Sebastian W, Samaranch L, Heller G, Kells A P, Bringas J, Pivirotto P, Forsayeth J, Bankiewicz K S (2013). Adeno-associated virus type 6 is retrogradely transported in the non-human primate brain. Gene Therapy.

[68] Masamizu Y., Okada T., Kawasaki K., Ishibashi H., Yuasa S., Takeda S., Hasegawa I., Nakahara K. (2011). Local and retrograde gene transfer into primate neuronal pathways via adeno-associated virus serotype 8 and 9. Neuroscience.

[69] Paz Jeanne T, Bryant Astra S, Peng Kathy, Fenno Lief, Yizhar Ofer, Frankel Wayne N, Deisseroth Karl, Huguenard John R (2011). A new mode of corticothalamic transmission revealed in the Gria4−/− model of absence epilepsy. Nature Neuroscience.

[70] Strick, P. L. & Kim, C. C. Input to primate motor cortex from posterior parietal cortex (area 5). I. Demonstration by retrograde transport. *Brain research* **157**, 325–330, ISSN: 0006–8993 (1978). 10.1016/0006-8993(78)90035-5 102409

[71] Zarzecki, P., Strick, P. L. & Asanuma, H. Input to primate motor cortex from posterior parietal cortex (area 5): Identification by antidromic activation. *Brain research* **157**, 331–335, ISSN: 0006–8993 (1978). 10.1016/0006-8993(78)90036-7 102410

[72] Lucier, G. E., Rüegg, D. C. & Wiesendanger, M. Responses of neurones in motor cortex and in area 3A to controlled stretches of forelimb muscles in cebus monkeys, en. *The Journal of physiology* **251**, 833–853, ISSN: 0022–3751 (1975). 10.1113/jphysiol.1975.sp011125 PMC1348420 127038

[73] Jones, E. G., Coulter, J. D. & Hendry, S. H. Intracortical connectivity of architectonic fields in the somatic sensory, motor and parietal cortex of monkeys. *The Journal of comparative neurology* **181**, 291–347, ISSN: 0021–9967 (1978). 10.1002/cne.901810206 99458

[74] Dum, R. P. & Strick, P. L. The Origin of Corticospinal Projections from the Premotor Areas in the Frontal Lobe. *The Journal of neuroscience*: *the official journal of the Society for Neuroscience* **11**, 667, ISSN: 0270–6474 (1991). 10.1523/JNEUROSCI.11-03-00667.1991 PMC6575356 1705965

[75] Dum, R. P. & Strick, P. L. Motor areas in the frontal lobe of the primate. *Physiology & behavior* **77**, 677–682, ISSN: 0031–9384 (2002). 10.1016/s0031-9384(02)00929-0 12527018

[76] López-Bendito, G. & Molnár, Z. Thalamocortical development: how are we going to get there? en. *Nature reviews*. *Neuroscience* **4**, 276–289, ISSN: 1471–003X, 1471–0048 (2003). 10.1038/nrn1075 12671644

[77] Bartlett, J. S., Wilcher, R. & Samulski, R. J. Infectious entry pathway of adeno-associated virus and adeno-associated virus vectors, en. *Journal of virology* **74**, 2777–2785, ISSN: 0022–538X (2000). 10.1128/jvi.74.6.2777-2785.2000 PMC111768 10684294

[78] Summerford, C. & Samulski, R. J. Membrane-associated heparan sulfate proteoglycan is a receptor for adeno-associated virus type 2 virions, en. *Journal of virology* **72**, 1438–1445, ISSN: 0022–538X (1998). 10.1128/jvi.72.2.1438-1445.1998 PMC124624 9445046

[79] Yoshihara Yoshihiro, Mizuno Takeo, Nakahira Masakiyo, Kawasaki Miwa, Watanabe Yasuyoshi, Kagamiyama Hiroyuki, Jishage Kou-ichi, Ueda Otoya, Suzuki Hiroshi, Tabuchi Katsuhiko, Sawamoto Kazunobu, Okano Hideyuki, Noda Tetsuo, Mori Kensaku (1999). A Genetic Approach to Visualization of Multisynaptic Neural Pathways Using Plant Lectin Transgene. Neuron.

[80] Horowitz, L. F., Montmayeur, J. P., Echelard, Y. & Buck, L. B. A genetic approach to trace neural circuits, en. *Proceedings of the National Academy of Sciences of the United States of America* **96**, 3194–3199, ISSN: 0027–8424 (1999). 10.1073/pnas.96.6.3194 PMC15918 10077660

[81] Maskos, U., Kissa, K., St Cloment, C. & Brûlet, P. Retrograde trans-synaptic transfer of green fluorescent protein allows the genetic mapping of neuronal circuits in transgenic mice. en. *Proceedings of the National Academy of Sciences of the United States of America* **99**, 10120–10125, ISSN: 0027–8424 (2002). 10.1073/pnas.152266799 PMC126634 12114537

[82] Luo Liqun, Callaway Edward M., Svoboda Karel (2008). Genetic Dissection of Neural Circuits. Neuron.

[83] Fenno Lief E, Mattis Joanna, Ramakrishnan Charu, Hyun Minsuk, Lee Soo Yeun, He Miao, Tucciarone Jason, Selimbeyoglu Aslihan, Berndt Andre, Grosenick Logan, Zalocusky Kelly A, Bernstein Hannah, Swanson Haley, Perry Chelsey, Diester Ilka, Boyce Frederick M, Bass Caroline E, Neve Rachael, Huang Z Josh, Deisseroth Karl (2014). Targeting cells with single vectors using multiple-feature Boolean logic. Nature Methods.

[84] Ohayon, S., Grimaldi, P., Schweers, N. & Tsao, D. Y. Saccade modulation by optical and electrical stimulation in the macaque frontal eye field, en. *The Journal of neuroscience*: *the official journal of the Society for Neuroscience* **33**, 16684–16697, ISSN: 0270–6474, 1529–2401 (2013). 10.1523/JNEUROSCI.2675-13.2013 PMC3797379 24133271

[85] Cavanaugh James, Monosov Ilya E., McAlonan Kerry, Berman Rebecca, Smith Mitchell K., Cao Vania, Wang Kuan H., Boyden Edward S., Wurtz Robert H. (2012). Optogenetic Inactivation Modifies Monkey Visuomotor Behavior. Neuron.

[86] Gerits, A., Farivar, R. & Rosen, B. R. Optogenetically Induced Behavioral and Functional Network Changes in Primates. *Current biology*: *CB* 1–5, ISSN: 0960–9822 (2012). 10.1016/j.cub.2012.07.023 PMC3461112 22840516

[87] Gold S. J., Hoang C. V., Potts B. W., Porras G., Pioli E., Kim K. W., Nadjar A., Qin C., LaHoste G. J., Li Q., Bioulac B. H., Waugh J. L., Gurevich E., Neve R. L., Bezard E. (2007). RGS9 2 Negatively Modulates L-3,4-Dihydroxyphenylalanine-Induced Dyskinesia in Experimental Parkinson's Disease. Journal of Neuroscience.

[88] Oyibo, H., Znamenskiy, P., Oviedo, H. V., Enquist, L. & Zador, A. Long-term Cre-mediated Retrograde Tagging of Neurons Using a Novel Recombinant Pseudorabies Virus. *Frontiers in neuroanatomy* **8**, 1–11, ISSN: 1662–5129 (2014). 10.3389/fnana.2014.00086 PMC4153299 25232307

[89] Soudais, C., Skander, N. & Kremer, E. J. Long-term *in vivo* transduction of neurons throughout the rat CNS using novel helper-dependent CAV-2 vectors. *FASEB journal*: *official publication of the Federation of American Societies for Experimental Biology* **18**, 391–393, ISSN: 0892–6638, 1530–6860 (2004). 10.1096/fj.03-0438fje 14688208

[90] Ni Jianguang, Wunderle Thomas, Lewis Christopher Murphy, Desimone Robert, Diester Ilka, Fries Pascal (2016). Gamma-Rhythmic Gain Modulation. Neuron.

[91] Benhamou, L., Bronfeld, M., Bar-Gad, I. & Cohen, D. Globus Pallidus external segment neuron classification in freely moving rats: a comparison to primates. *PloS one* **7**, e45421, ISSN: 1932–6203 (2012). 10.1371/journal.pone.0045421 PMC3448641 23028997

[92] Gunaydin Lisa A, Yizhar Ofer, Berndt André, Sohal Vikaas S, Deisseroth Karl, Hegemann Peter (2010). Ultrafast optogenetic control. Nature Neuroscience.

[93] Fenno, L., Yizhar, O. & Deisseroth, K. The development and application of optogenetics. *Annual review of neuroscience* **34**, 389–412, ISSN: 0147–006X, 1545–4126 (2011). 10.1146/annurev-neuro-061010-113817 PMC6699620 21692661

[94] Deisseroth, K. Optogenetics. *Nature methods* 1–4, ISSN: 1548–7091 (2011). 10.1038/nmeth.f.324 PMC6814250 21191368

[95] Deisseroth, K. & Schnitzer, M. J. Engineering approaches to illuminating brain structure and dynamics. *Neuron* **80**, 568–577, ISSN: 0896–6273, 1097–4199 (2013). 10.1016/j.neuron.2013.10.032 PMC5731466 24183010

[96] Thomas, R. K. & Boyd, M. G. A comparison of Cebus albifrons and Saimiri sciureus on oddity performance. *Animal learning & behavior*, ISSN: 0090–4996 (1973).

[97] Overman, W. H., McLain, C, Ormsby, G. E. & Brooks, V. Visual recognition memory in squirrel monkeys. *Animal learning & behavior* **11**, 483–488, ISSN: 0090–4996 (1983).

[98] Hudson, R., Laska, M. & Ploog, D. A New Method for Testing Perceptual and Learning Capacities in Unrestrained Small Primates. *Folia primatologica; international journal of primatology*, ISSN: 00155713 (1992). 10.1159/000156643 1473782

[99] Laska, M., Alicke, T. & Hudson, R. A study of long-term odor memory in squirrel monkeys (Saimiri sciureus). en. *Journal of comparative psychology* **110**, 125–130, ISSN: 0093–4127 (1996). 10.1037/0735-7036.110.2.125 8681526

[100] De Valois, R. L. & Morgan, H. C. Psychophysical studies of monkey vision. II. Squirrel monkey wavelength and saturation discrimination. *Vision research* **14**, 69–73, ISSN: 0042–6989 (1974). 10.1016/0042-6989(74)90117-5 4204838

[101] Kelleher, R. T., Gill, C. A., Riddle, W. C. & Cook, L. On the use of the squirrel monkey in behavioral and pharmacological experiments. *Journal of the experimental analysis of behavior* **6**, 249–252, ISSN: 0022–5002 (1963). 10.1901/jeab.1963.6-249 PMC1404291 14031746

[102] Barrett James E. (1985). Behavioral Pharmacology of the Squirrel Monkey. Handbook of Squirrel Monkey Research.

[103] Katz, J. L., Terry, P. & Witkin, J. M. Comparative behavioral pharmacology and toxicology of cocaine and its ethanol-derived metabolite, cocaine ethyl-ester (cocaethylene). *Life sciences* **50**, 1351–1361, ISSN: 0024–3205 (1992). 10.1016/0024-3205(92)90286-x 1532847

[104] Czoty, P. W., Justice, J. B. & Howell, L. L. Cocaine-induced changes in extracellular dopamine determined by microdialysis in awake squirrel monkeys. *Psychopharmacology* **148**, 299–306, ISSN: 0033–3158 (2000). 10.1007/s002130050054 10755743

[105] Lyons, D. M. & Levine, S. Socioregulatory effects on squirrel monkey pituitary-adrenal activity: a longitudinal analysis of Cortisol and ACTH. *Psychoneuroendocrinology* **19**, 283–291, ISSN: 0306–4530 (1994). 10.1016/0306-4530(94)90066-3 8202576

[106] Levine, S., Lyons, D. M. & Schatzberg, A. F. Psychobiological consequences of social relationships. *Annals of the New York Academy of Sciences* **807**, 210–218, ISSN: 0077–8923 (1997). 10.1111/j.1749-6632.1997.tb51922.x 9071353

[107] Lyons, D. M., Parker, K. J. & Schatzberg, A. F. Animal models of early life stress: implications for understanding resilience. *Developmental psychobiology* **52**, 616–624, ISSN: 0012–1630, 1098–2302 (2010). 10.1002/dev.20500 PMC6716163 20957724

[108] Lyons D. M., Buckmaster P. S., Lee A. G., Wu C., Mitra R., Duffey L. M., Buckmaster C. L., Her S., Patel P. D., Schatzberg A. F. (2010). Stress coping stimulates hippocampal neurogenesis in adult monkeys. Proceedings of the National Academy of Sciences.

[109] Chambers, J. K., Kuribayashi, H., Ikeda, S.-I. & Une, Y. Distribution of neprilysin and deposit patterns of Abeta subtypes in the brains of aged squirrel monkeys (Saimiri sciureus), en. *Amyloid*: *the international journal of experimental and clinical investigation*: *the official journal of the International Society of Amyloidosis* **17**, 75–82, ISSN: 1350–6129, 1744–2818 (2010). 10.3109/13506129.2010.483119 20462366

[110] Langston, J. W., Forno, L. S., Rebert, C. S. & Irwin, I. Selective nigral toxicity after systemic administration of l-methyl-4-phenyl-l,2,5,6-tetrahydropyrine (MPTP) in the squirrel monkey. *Brain research* **292**, 390–394, ISSN: 0006–8993 (1984). 10.1016/0006-8993(84)90777-7 6607092

[111] Brady A. G. (2000). Research Techniques for the Squirrel Monkey (Saimiri sp.). ILAR Journal.

[112] Gao, Y. *et al*. A brain MRI atlas of the common squirrel monkey, Saimiri sciureus. *Proceedings* -*Society of Photo*-*Optical Instrumentation Engineers* **9038**, 90380C, ISSN: 1018–4732 (2014). 10.1117/12.2043589 PMC4013108 24817811

[113] Gergen, J. A. & MacLean, P. D. *A Stereotaxic Atlas of the Squirrel Monkey’s Brain* (Gergen JA; MacLean PD (1962) A Stereotaxic Atlas of the Squirrel Monkey's Brain. Public Health Service Publication No 933. Bethesda, MD: NIH., 1962).

[114] Emmers, R. & Akert, K. *A stereotaxic atlas of the brain of the squirrel monkey* (*Saimiri sciureus*) (Univ. of Wisconsin Press, Madison, 1963).

[115] Luan, H., Gdowski, M. J., Newlands, S. D. & Gdowski, G. T. Convergence of vestibular and neck proprioceptive sensory signals in the cerebellar interpositus. *The Journal of neuroscience*: *the official journal of the Society for Neuroscience* **33**, 1198–210a, ISSN: 0270–6474, 1529–2401 (2013). 10.1523/JNEUROSCI.3460-12.2013 PMC3711745 23325256

[116] Lewis, R. F., Haburcakova, C., Gong, W., Karmali, F. & Merfeld, D. M. Spatial and temporal properties of eye movements produced by electrical stimulation of semicircular canal afferents. *Journal of neurophysiology* **108**, 1511–1520, ISSN: 0022–3077, 1522–1598 (2012). 10.1152/jn.01029.2011 PMC3544955 22673321

[117] Igarashi, M. & Kato, Y. Effect of different vestibular lesions upon body equilibrium function in squirrel monkeys. *Acta oto*-*laryngologica*. *Supplementum* **330**, 91–99, ISSN: 0365–5237 (1975). 10.3109/00016487509121280 811080

[118] Kleinlogel, H. Sleep in various species of laboratory animals. *Neuropsychobiology* **9**, 174–177, ISSN: 0302–282X (1983). 10.1159/000117959 6621856

[119] Malone, B. J., Heiser, M. A., Beitel, R. E. & Schreiner, C. E. Background noise exerts diverse effects on the cortical encoding of foreground sounds, en. *Journal of neurophysiology* **118**, 1034–1054, ISSN: 0022–3077, 1522–1598 (2017). 10.1152/jn.00152.2017 PMC5547268 28490644

[120] Cheung Steven W. (2005). Frequency Map Variations in Squirrel Monkey Primary Auditory Cortex. The Laryngoscope.

[121] Jürgens, U., Maurus, M., Ploog, D. & Winter, P. Vocalization in the squirrel monkey (Saimiri sciureus) elicited by brain stimulation. *Experimental brain research*. *Experimentelle Hirnforschung*. *Experimentation cerebrale* **4**, 114–117, ISSN: 0014–4819 (1967). 10.1007/BF00240356 4970751

[122] Pieper, F. & Jürgens, U. Neuronal activity in the inferior colliculus and bordering structures during vocalization in the squirrel monkey. *Brain research* **979**, 153–164, ISSN: 0006–8993 (2003). 10.1016/s0006-8993(03)02897-x 12850582

[123] Düsterhöft, F., Häusler, U. & Jürgens, U. Neuronal activity in the periaqueductal gray and bordering structures during vocal communication in the squirrel monkey. *Neuroscience* **123**, 53–60, ISSN: 0306–4522 (2004). 10.1016/j.neuroscience.2003.07.007 14667441

[124] Lemon, R. N., Kirkwood, P. A., Maier, M. A., Nakajima, K. & Nathan, P. Direct and indirect pathways for corticospinal control of upper limb motoneurons in the primate. *Progress in brain research* **143**, 263–279, ISSN: 0079–6123 (2004). 10.1016/S0079-6123(03)43026-4 14653171

[125] Négyessy László, Pálfi Emese, Ashaber Mária, Palmer Cory, Jákli Balázs, Friedman Robert M., Chen Li M., Roe Anna W. (2013). Intrinsic horizontal connections process global tactile features in the primary somatosensory cortex: Neuroanatomical evidence. Journal of Comparative Neurology.

[126] Chen, L. M., Friedman, R. M. & Roe, A. W. Optical imaging of digit topography in individual awake and anesthetized squirrel monkeys, en. *Experimental brain research*. *Experimentelle Hirnforschung*. *Experimentation cerebrale* **196**, 393–401, ISSN: 0014–4819, 1432–1106 (2009). 10.1007/s00221-009-1861-y PMC3786732 19484466

[127] Friedman, R. M., Chen, L. M. & Roe, A. W. Responses of areas 3b and 1 in anesthetized squirrel monkeys to single- and dual-site stimulation of the digits, en. *Journal of neurophysiology* **100**, 3185–3196, ISSN: 0022–3077 (2008). 10.1152/jn.90278.2008 PMC2604853 18922955

[128] Sur, M., Nelson, R. J. & Kaas, J. H. Representations of the body surface in cortical areas 3b and 1 of squirrel monkeys: comparisons with other primates, en. *The Journal of comparative neurology* **211**, 177–192, ISSN: 0021–9967 (1982). 10.1002/cne.902110207 7174889

[129] Bortoff, G. A. & Strick, P. L. Corticospinal terminations in two new-world primates: further evidence that corticomotoneuronal connections provide part of the neural substrate for manual dexterity. *The Journal of neuroscience*: *the official journal of the Society for Neuroscience* **13**, 5105–5118, ISSN: 0270–6474 (1993). 10.1523/JNEUROSCI.13-12-05105.1993 PMC6576412 7504721

[130] Donoghue, J. P., Leibovic, S. & Sanes, J. N. Organization of the forelimb area in squirrel monkey motor cortex: representation of digit, wrist, and elbow muscles. *Experimental brain research*. *Experimentelle Hirnforschung*. *Experimentation cerebrale* 1–19, ISSN: 0014–4819 (1992). 10.1007/BF00228996 1601087

[131] Dancause Numa, Barbay Scott, Frost Shawn B., Plautz Erik J., Stowe Ann M., Friel Kathleen M., Nudo Randolph J. (2006). Ipsilateral connections of the ventral premotor cortex in a new world primate. The Journal of Comparative Neurology.

[132] Dancause N., Duric V., Barbay S., Frost S. B., Stylianou A., Nudo R. J. (2008). An Additional Motor-Related Field in the Lateral Frontal Cortex of Squirrel Monkeys. Cerebral Cortex.

[133] Omrani, M., Kaufman, M. T., Hatsopoulos, N. G. & Cheney, P. D. Perspectives on classical controversies about the motor cortex, en. *Journal of neurophysiology* **118**, 1828–1848, ISSN: 0022–3077, 1522–1598 (2017). 10.1152/jn.00795.2016 PMC5599665 28615340

[134] Scott Stephen H. (2016). A Functional Taxonomy of Bottom-Up Sensory Feedback Processing for Motor Actions. Trends in Neurosciences.

[135] Nudo R. J., Larson D., Plautz E. J., Friel K. M., Barbay S., Frost S. B. (2003). A Squirrel Monkey Model of Poststroke Motor Recovery. ILAR Journal.

[136] Eisner-Janowicz Ines, Barbay Scott, Hoover Erica, Stowe Ann M., Frost Shawn B., Plautz Erik J., Nudo Randolph J. (2008). Early and Late Changes in the Distal Forelimb Representation of the Supplementary Motor Area After Injury to Frontal Motor Areas in the Squirrel Monkey. Journal of Neurophysiology.

[137] Barbay Scott, Plautz Erik J., Friel Kathleen M., Frost Shawn B., Dancause Numa, Stowe Ann M., Nudo Randolph J. (2005). Behavioral and neurophysiological effects of delayed training following a small ischemic infarct in primary motor cortex of squirrel monkeys. Experimental Brain Research.

[138] Friel Kathleen M., Barbay Scott, Frost Shawn B., Plautz Erik J., Stowe Ann M., Dancause Numa, Zoubina Elena V., Nudo Randolph J. (2007). Effects of a Rostral Motor Cortex Lesion on Primary Motor Cortex Hand Representation Topography in Primates. Neurorehabilitation and Neural Repair.

[139] Friel Kathleen M., Barbay Scott, Frost Shawn B., Plautz Erik J., Hutchinson Douglas M., Stowe Ann M., Dancause Numa, Zoubina Elena V., Quaney Barbara M., Nudo Randolph J. (2005). Dissociation of Sensorimotor Deficits After Rostral Versus Caudal Lesions in the Primary Motor Cortex Hand Representation. Journal of Neurophysiology.

[140] Reed, J. L., Liao, C.-C, Qi, H.-X. & Kaas, J. H. Plasticity and Recovery After Dorsal Column Spinal Cord Injury in Nonhuman Primates, en. *Journal of experimental neuroscience* **10**, 11–21, ISSN: 1179–0695 (2016). 10.4137/JEN.S40197 PMC4991577 27578996

[141] Liao, C.-C, Reed, J. L., Kaas, J. H. & Qi, H.-X. Intracortical connections are altered after longstanding deprivation of dorsal column inputs in the hand region of area 3b in squirrel monkeys, en. *The Journal of comparative neurology* **524**, 1494–1526, ISSN: 0021–9967, 1096–9861 (2016). 10.1002/cne.23921 PMC4783257 26519356

[142] Qi, H.-X., Reed, J. L., Gharbawie, O. A., Burish, M. J. & Kaas, J. H. Cortical neuron response properties are related to lesion extent and behavioral recovery after sensory loss from spinal cord injury in monkeys, en. *The Journal of neuroscience*: *the official journal of the Society for Neuroscience* **34**, 4345–4363, ISSN: 0270–6474, 1529–2401 (2014). 10.1523/JNEUROSCI.4954-13.2014 PMC3960473 24647955

[143] Qi, H.-X., Kaas, J. H. & Reed, J. L. The reactivation of somatosensory cortex and behavioral recovery after sensory loss in mature primates, en. *Frontiers in systems neuroscience* **8**, 84, ISSN: 1662–5137 (2014). 10.3389/fnsys.2014.00084 PMC4026759 24860443

[144] Han Xue (2012). Optogenetics in the nonhuman primate. Progress in Brain Research.

[145] Deng, C., Yuan, H. & Dai, J. Behavioral Manipulation by Optogenetics in the Nonhuman Primate, en. *The Neuroscientist*: *a review journal bringing neurobiology*, *neurology and psychiatry* 1073858417728459, ISSN: 1073–8584, 1089–4098 (2017). 10.1177/1073858417728459 28874078

[146] Kishi, N., Sato, K., Sasaki, E. & Okano, H. Common marmoset as a new model animal for neuroscience research and genome editing technology. *Development*, *growth & differentiation* **56**, 53–62, ISSN: 0012–1592, 1440–169X (2014). 10.1111/dgd.12109 24387631

[147] Sasaki Erika (2015). Prospects for genetically modified non-human primate models, including the common marmoset. Neuroscience Research.

[148] Okano, H., Hikishima, K., Iriki, A. & Sasaki, E. The common marmoset as a novel animal model system for biomedical and neuroscience research applications. *Seminars in fetal & neonatal medicine* **17**, 336–340, ISSN: 1744–165X, 1878–0946 (2012). 10.1016/j.siny.2012.07.002 22871417

[149] Sasaki Erika, Suemizu Hiroshi, Shimada Akiko, Hanazawa Kisaburo, Oiwa Ryo, Kamioka Michiko, Tomioka Ikuo, Sotomaru Yusuke, Hirakawa Reiko, Eto Tomoo, Shiozawa Seiji, Maeda Takuji, Ito Mamoru, Ito Ryoji, Kito Chika, Yagihashi Chie, Kawai Kenji, Miyoshi Hiroyuki, Tanioka Yoshikuni, Tamaoki Norikazu, Habu Sonoko, Okano Hideyuki, Nomura Tatsuji (2009). Generation of transgenic non-human primates with germline transmission. Nature.

[150] Hsu, P. D., Lander, E. S. & Zhang, F. Development and applications of CRISPR-Cas9 for genome engineering, en. *Cell* **157**, 1262–1278, ISSN: 0092–8674, 1097–4172 (2014). 10.1016/j.cell.2014.05.010 PMC4343198 24906146

[151] Mitchell, J. F., Reynolds, J. H. & Miller, C. T. Active vision in marmosets: a model system for visual neuroscience. *The Journal of neuroscience*: *the official journal of the Society for Neuroscience* **34**, 1183–1194, ISSN: 0270–6474, 1529–2401 (2014). 10.1523/JNEUROSCI.3899-13.2014 PMC3898283 24453311

[152] Mitchell, J. F. & Leopold, D. A. The marmoset monkey as a model for visual neuroscience. *Neuroscience research* **93**, 20–46, ISSN: 0168–0102 (2015). 10.1016/j.neures.2015.01.008 PMC4408257 25683292

[153] Okano, H. & Mitra, P. Brain-mapping projects using the common marmoset. *Neuroscience research* **93**, 3–7, ISSN: 0168–0102 (2015). 10.1016/j.neures.2014.08.014 25264372

[154] Bakola, S., Burman, K. J. & Rosa, M. G. P. The cortical motor system of the marmoset monkey (Callithrix jacchus). *Neuroscience research* **93**, 72–81, ISSN: 0168–0102 (2015). 10.1016/j.neures.2014.11.003 25498953

[155] Burkart, J. M. & Finkenwirth, C. Marmosets as model species in neuroscience and evolutionary anthropology. *Neuroscience research* **93**, 8–19, ISSN: 0168–0102 (2014). 10.1016/j.neures.2014.09.003 25242577

[156] Dea, M., Hamadjida, A., Elgbeili, G., Quessy, S. & Dancause, N. Different Patterns of Cortical Inputs to Subregions of the Primary Motor Cortex Hand Representation in Cebus apella. en. *Cerebral cortex* **26**, 1747–1761, ISSN: 1047–3211, 1460–2199 (2016). 10.1093/cercor/bhv324 PMC4785954 26966266

[157] Young, N. A., Collins, C. E. & Kaas, J. H. Cell and neuron densities in the primary motor cortex of primates, en. *Frontiers in neural circuits* **7**, 30, ISSN: 1662–5110 (2013). 10.3389/fncir.2013.00030 PMC3583034 23450743

[158] Liao, C.-C, Gharbawie, O. A., Qi, H. & Kaas, J. H. Cortical connections to single digit representations in area 3b of somatosensory cortex in squirrel monkeys and prosimian galagos. en. *The Journal of comparative neurology* **521**, 3768–3790, ISSN: 0021–9967, 1096–9861 (2013). 10.1002/cne.23377 PMC4000754 23749740

[159] Qi, H.-X., Gharbawie, O. A., Wong, P. & Kaas, J. H. Cell-poor septa separate representations of digits in the ventroposterior nucleus of the thalamus in monkeys and prosimian galagos. *The Journal of comparative neurology* **519**, 738–758, ISSN: 0021–9967, 1096–9861 (2011). 10.1002/cne.22545 PMC3695620 21246552

[160] Dancause Numa, Barbay Scott, Frost Shawn B., Plautz Erik J., Popescu Mihai, Dixon Philip M., Stowe Ann M., Friel Kathleen M., Nudo Randolph J. (2005). Topographically Divergent and Convergent Connectivity between Premotor and Primary Motor Cortex. Cerebral Cortex.

[161] Heffner, R. S. & Masterton, R. B. The role of the corticospinal tract in the evolution of human digital dexterity, en. *Brain*, *behavior and evolution* **23**, 165–183, ISSN: 0006–8977 (1983). 10.1159/000121494 6667369

[162] Qi Hui-Xin, Reed Jamie L., Franca Joao G., Jain Neeraj, Kajikawa Yoshinao, Kaas Jon H. (2016). Chronic recordings reveal tactile stimuli can suppress spontaneous activity of neurons in somatosensory cortex of awake and anesthetized primates. Journal of Neurophysiology.

[163] Nakajima, K., Maier, M. A., Kirkwood, P. A. & Lemon, R. N. Striking differences in transmission of corticospinal excitation to upper limb motoneurons in two primate species, en. *Journal of neurophysiology* **84**, 698–709, ISSN: 0022–3077 (2000). 10.1152/jn.2000.84.2.698 10938297

[164] Maier M. A., Olivier E., Baker S. N., Kirkwood P. A., Morris T., Lemon R. N. (1997). Direct and Indirect Corticospinal Control of Arm and Hand Motoneurons in the Squirrel Monkey (Saimiri sciureus). Journal of Neurophysiology.

[165] Lemon Roger (2004). Cortico-motoneuronal system and dexterous finger movements. Journal of Neurophysiology.

[166] Kinoshita Masaharu, Matsui Ryosuke, Kato Shigeki, Hasegawa Taku, Kasahara Hironori, Isa Kaoru, Watakabe Akiya, Yamamori Tetsuo, Nishimura Yukio, Alstermark Bror, Watanabe Dai, Kobayashi Kazuto, Isa Tadashi (2012). Genetic dissection of the circuit for hand dexterity in primates. Nature.

[167] Ozden, I. *et al*. A coaxial optrode as multifunction write-read probe for optogenetic studies in non-human primates. *Journal of M euroscience methods* **219**, 142–154, ISSN: 1872–678X (2013). 10.1016/j.jneumeth.2013.06.011 PMC3789534 23867081

[168] Noudoost, B. & Moore, T. A reliable microinjectrode system for use in behaving monkeys. *Journal of neuroscience methods* **194**, 218–223, ISSN: 0165–0270 (2011). 10.1016/j.jneumeth.2010.10.009 PMC3161729 20951736

[169] Churchland, M. M. & Shenoy, K. V. Delay of movement caused by disruption of cortical preparatory activity, en. *Journal of neurophysiology* **97**, 348–359, ISSN: 0022–3077 (2007). 10.1152/jn.00808.2006 17005608

[170] Lyons, D. M., Yang, C, Eliez, S., Reiss, A. L. & Schatzberg, A. F. Cognitive correlates of white matter growth and stress hormones in female squirrel monkey adults. *The Journal of neuroscience*: *the official journal of the Society for Neuroscience* **24**, 3655–3662, ISSN: 0270–6474 (2004). 10.1523/JNEUROSCI.0324-04.2004 PMC6729742 15071114

[171] Bartzokis, G. *et al*. Age-related changes in frontal and temporal lobe volumes in men: a magnetic resonance imaging study. *Archives of general psychiatry* **58**, 461–465, ISSN: 0003–990X (2001). 10.1001/archpsyc.58.5.461 11343525

[172] Warden Melissa R., Selimbeyoglu Aslihan, Mirzabekov Julie J., Lo Maisie, Thompson Kimberly R., Kim Sung-Yon, Adhikari Avishek, Tye Kay M., Frank Loren M., Deisseroth Karl (2012). A prefrontal cortex–brainstem neuronal projection that controls response to behavioural challenge. Nature.

