# Publisher Correction: Development of an optogenetic toolkit for neural circuit dissection in squirrel monkeys

**DOI:** 10.1038/s41598-019-55025-w

**Published:** 2019-12-05

**Authors:** Daniel J. O’Shea, Paul Kalanithi, Emily A. Ferenczi, Brian Hsueh, Chandramouli Chandrasekaran, Werapong Goo, Ilka Diester, Charu Ramakrishnan, Matthew T. Kaufman, Stephen I. Ryu, Kristen W. Yeom, Karl Deisseroth, Krishna V. Shenoy

**Affiliations:** 1 Neurosciences Program, Stanford University, Stanford, CA USA; 2 Department of Electrical Engineering, Stanford University, Stanford, CA USA; 3 Department of Neurosurgery, Stanford University, Stanford, CA USA; 4 Department of Bioengineering, Stanford University, Stanford, CA USA; 5 Department of Psychiatry and Behavioral Science, Stanford University, Stanford, CA USA; 6 Department of Neurobiology, Stanford University, Stanford, CA USA; 7 Department of Radiology, Stanford University, Stanford, CA USA; 8 Department of Otophysiologie, Albert Ludwig University of Freiburg, Freiburg im Breisgau, Germany; 9 BrainLinks-BrainTools, Albert Ludwig University of Freiburg, Freiburg im Breisgau, Germany; 10 Cold Spring Harbor Laboratory, Cold Spring Harbor, NY USA; 11 Palo Alto Medical Foundation, Palo Alto, CA USA; 12 Howard Hughes Medical Institute, Stanford University, Stanford, CA USA

Correction to: *Scientific Reports* 10.1038/s41598-018-24362-7, published online 30 April 2018

In Figure 1, incorrect scale bars were present in the figure. The correct Figure 1 appears below.

**Figure 1:**
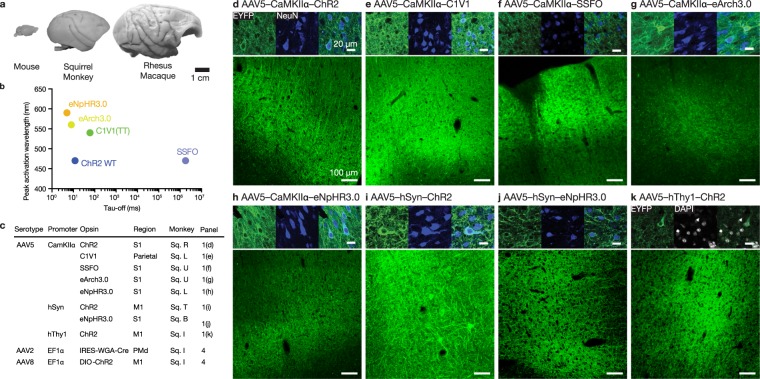
.

