# Supplementary material for: Development of an optogenetic toolkit for neural circuit dissection in squirrel monkeys
Source: Sci Rep. 2018 Apr 30;8:6775. doi: 10.1038/s41598-018-24362-7 (PMC5928036; doi:10.1038/s41598-018-24362-7)
Supplement: Supplementary file 1 — Supplementary Materials [file 41598_2018_24362_MOESM1_ESM.pdf]

Supplemental Materials

Development of an optogenetic toolkit for neural circuit dissection in squirrel monkeys

Daniel J. O'Shea[1,2,*,+], Paul Kalanithi[2,3,+], Emily Ferenczi[3], Brian Hsueh[4], Chandramouli Chandrasekaran[2], Werapong Goo[4], Ilka Diester[2,4,8,9], Charu Ramakrishnan[4], Matthew T. Kaufman[1,10], Stephen I Ryu[2,11], Kristen W. Yeom[7], Karl Deisseroth[4,5,12,†] and Krishna V. Shenoy[1,2,4,6,12,†]

[1]Neurosciences Program, [2]Department of Electrical Engineering, [3]Department of Neurosurgery, [4]Department of Bioengineering, [5]Department of Psychiatry and Behavioral Science, [6]Deparment of Neurobiology, [7]Department of Radiology, Stanford University, [8]Department of Otophysiologie, [9]BrainLinks-BrainTools, Albert Ludwig University of Freiburg, [10]Cold Spring Harbor Laboratory, [11]Palo Alto Medical Foundation, Palo Alto, CA, [12]Howard Hughes Medical Institute

[+,†] These authors contributed equally to this manuscript.

Supplemental Figures

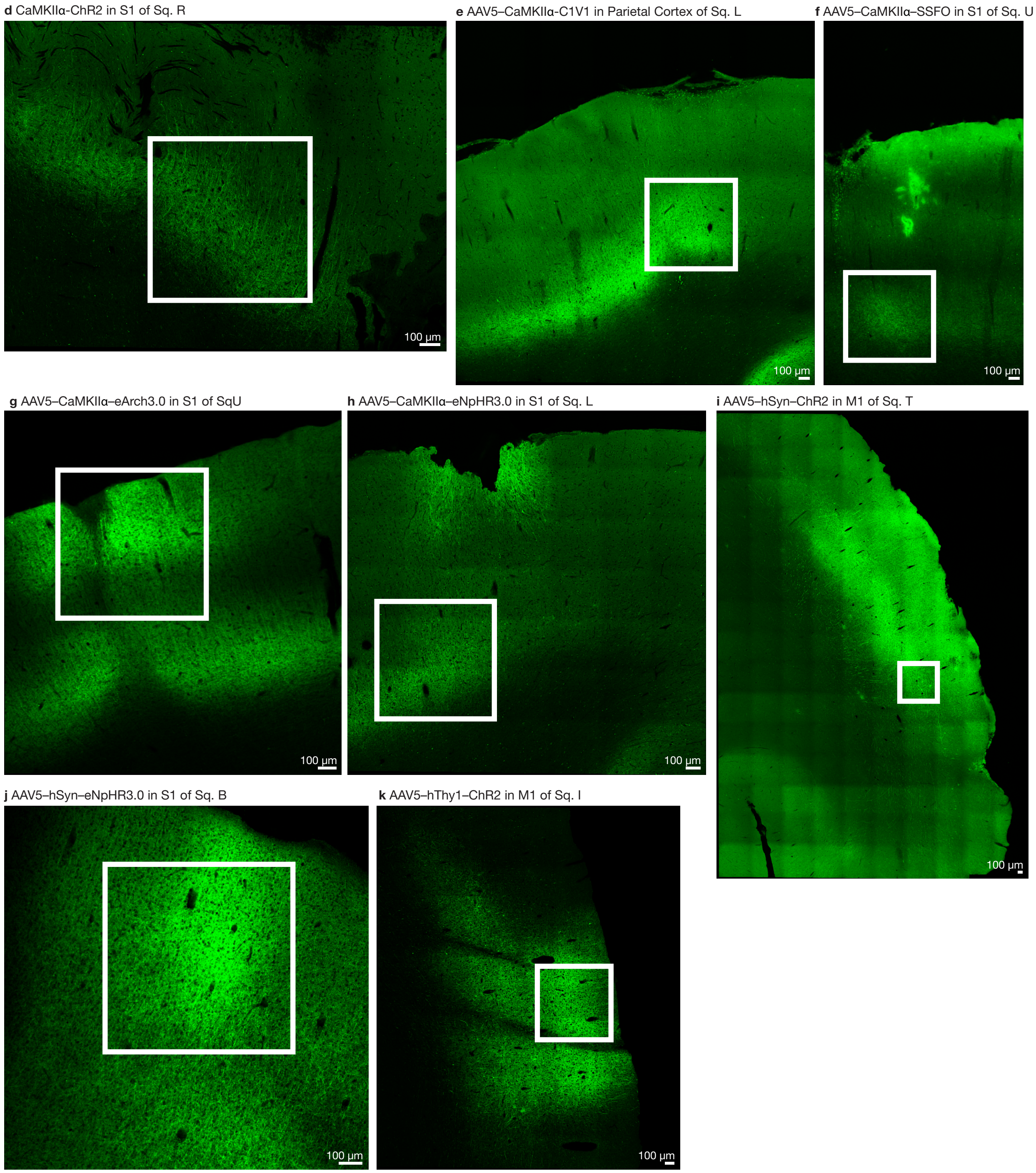

Supp. Figure 1: Larger views of squirrel monkey opsin expression panel. Larger views of the representative histology for first 8 opsins shown in Figure 1. Subpanel lettering corresponds to lettering in Figure 1. Viral construct, subject, and brain region are as indicated. White squares indicate the 800 µm region presented in Figure 1.

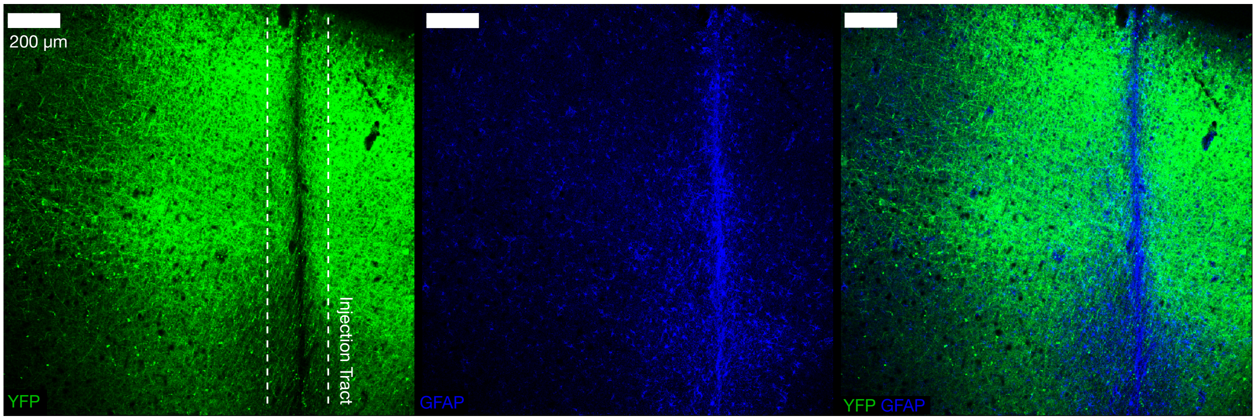

Supp. Figure 2: Minimal gliosis was observed in the vicinity of injection sites. Strong expression of AAV5-hSyn-ChR2(H134R)-EYFP (green) in Left M1 cortex of Sq. E was observed near the injection track. Glial fibrillary acidic protein (GFAP, blue) was observed only in the immediate vicinity of the injection track, indicating that the presence of reactive astrocytes and the formation of glial scars was confined to 50 μm from the injection site.

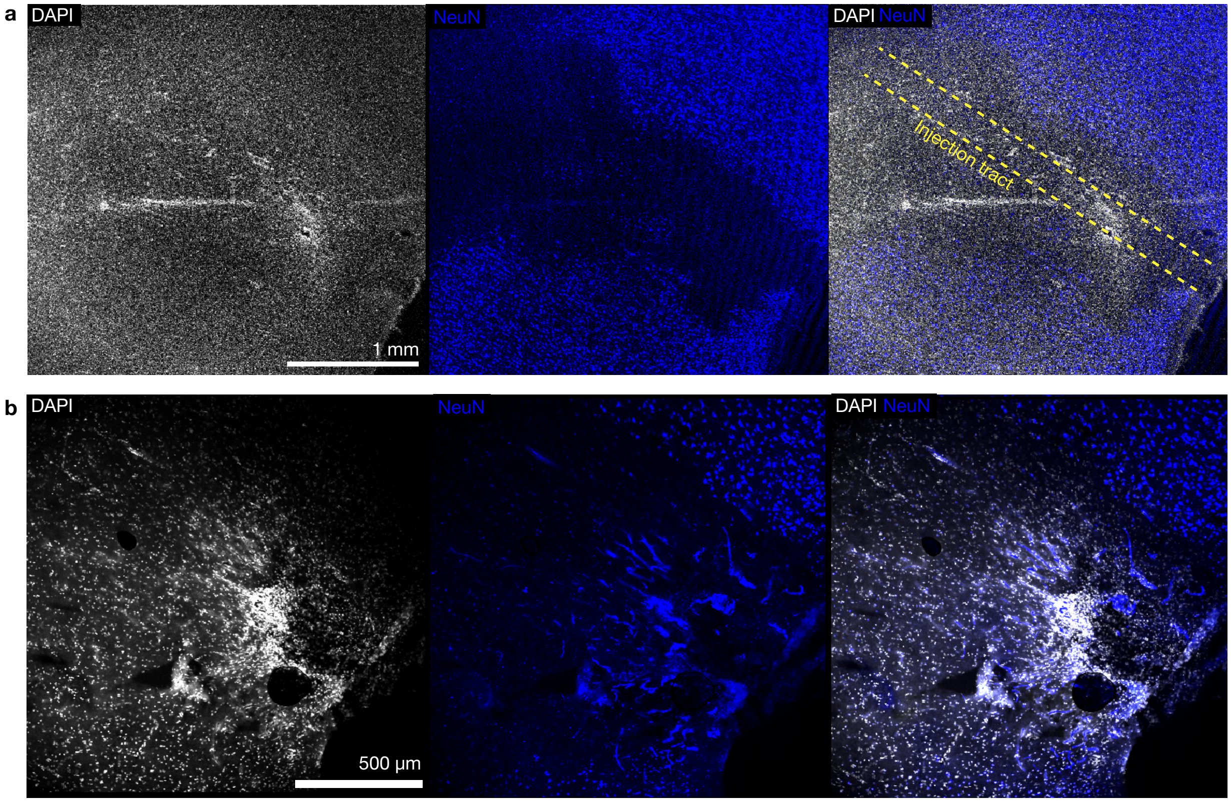

Supp. Figure 3: Neuronal cell death following injection of LT-HSV. Dual injection of LT-HSV-EF1α-Cre into M1 and AAV5-hSyn-DIO-EYFP into PMd of Sq. R as a projection targeting strategy caused cell death in M1 around the injection site. No expression of EYFP was observed in PMd. **(a)** 10x overview of M1 injection site. **(b)** 20x zoom of neuronal cell death.

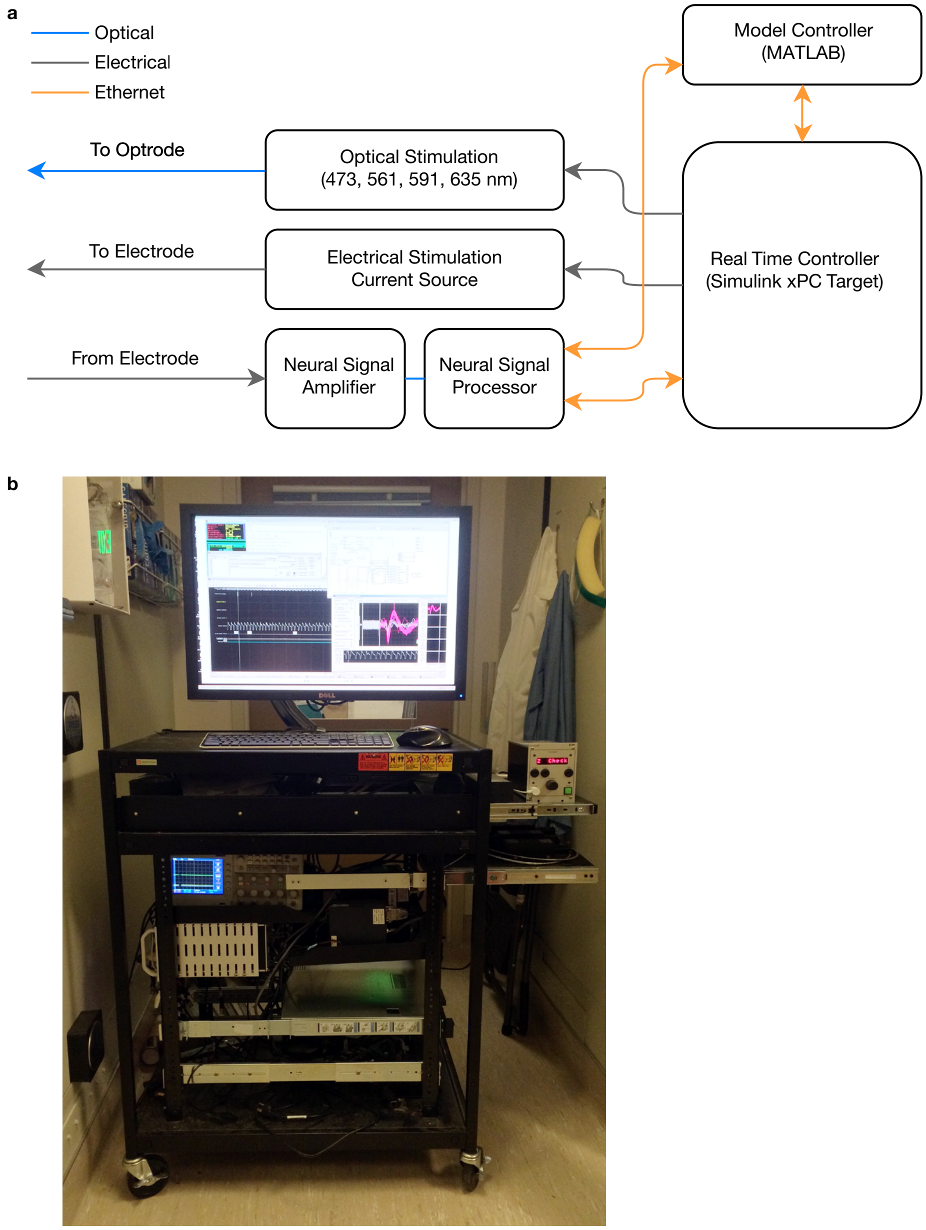

Supp. Figure 4: Real-time portable optical stimulation and electrophysiology cart. Schematic of integrated devices and connectivity (a) and photograph (b). This cart enabled multi-wavelength optical stimulation and low-noise electrical recording within an operating room setting in anesthetized squirrel monkeys.

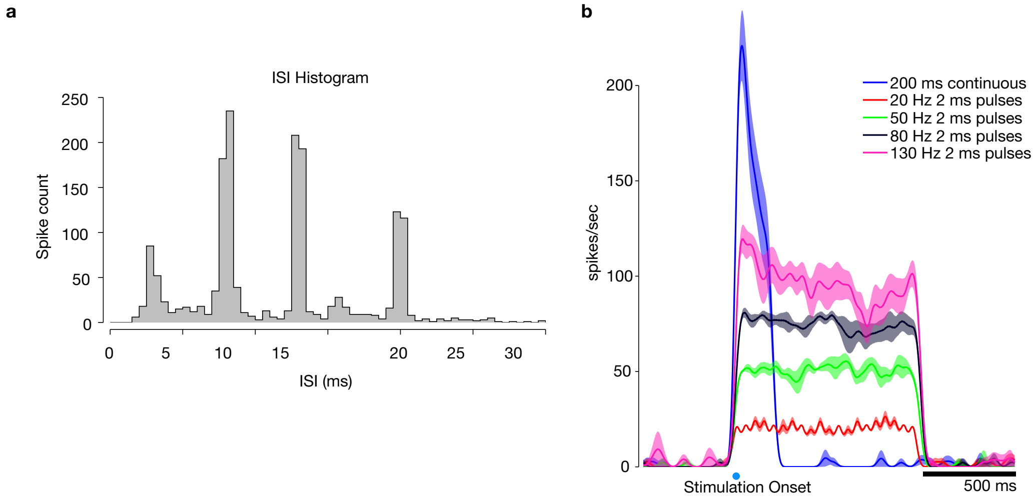

Supp. Figure 5: Inter-spike interval histogram and firing-rate of light-responsive presumed ChR2-expressing unit in Sq. P. This unit, also depicted in Figure 5a was recorded in left M1 cortex in Sq. P which was injected with AAV5-hSyn-ChR2(H134R)-EYFP. **(a)** Inter-spike interval (ISI) histogram for combined spontaneous and light-evoked spikes. We note that due to extremely low spontaneous firing rates under isofluorane anesthesia, the ISI histogram for spontaneous spikes is near zero due to the rarity of pairs of spikes within 35 ms. **(b)** Trial-averaged stimulation onset-aligned smoothed firing rate during each pattern of stimulation. Pulse trains were 1000 ms in duration; continuous pulses were 200 ms in duration.

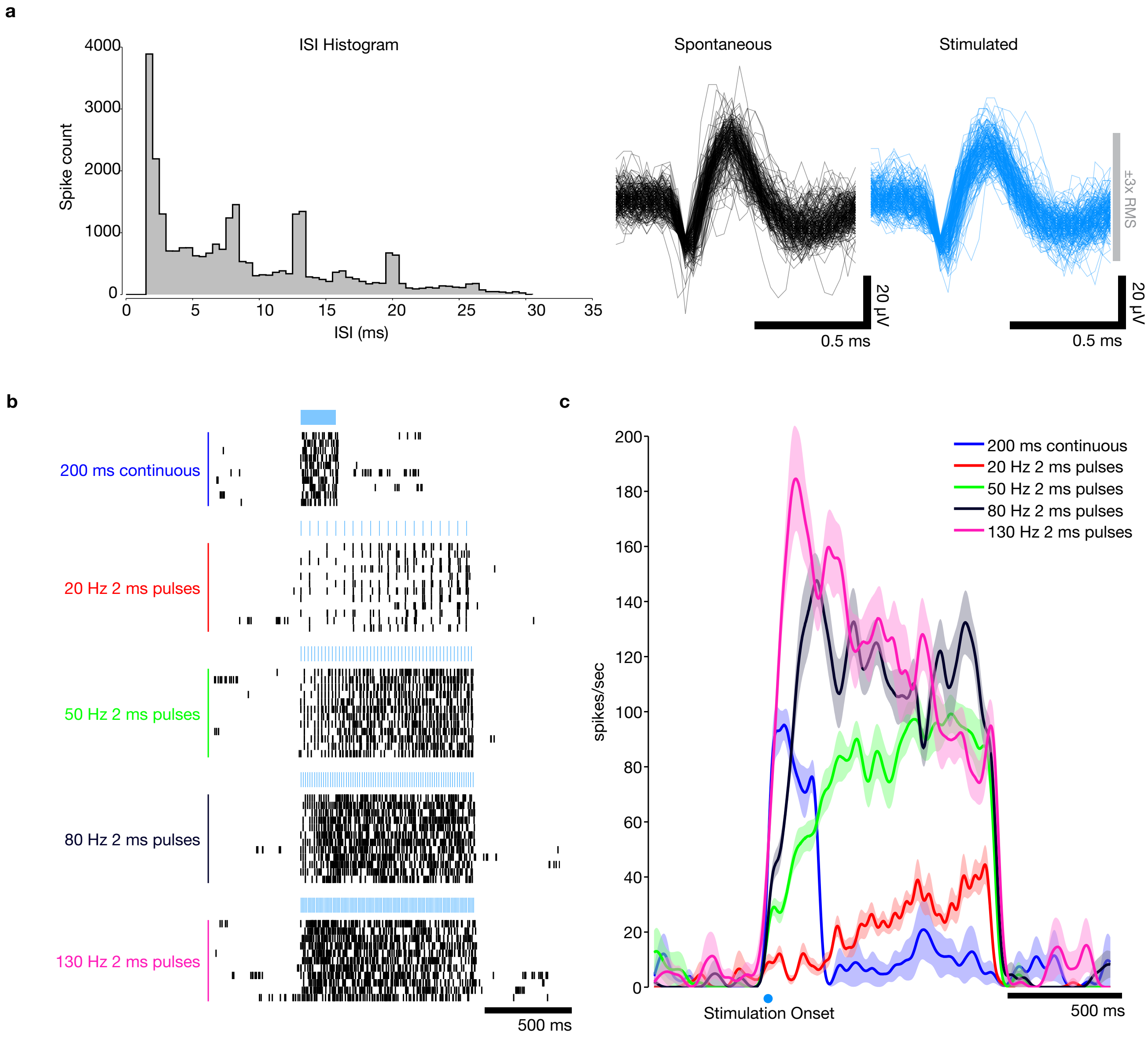

Supp. Figure 6: Response of a light-responsive presumed ChR2(H134R)-expressing single-unit in Sq. P. This neuron was recorded in left M1 cortex in Sq. P injected with AAV5-hSyn-ChR2(H134R)-EYFP. **(a)** Interspike interval histogram and sample spontaneous and light-evoked waveforms recorded for this unit. **(b)** Stimulation aligned spike raster during 1000 ms pulse trains of varying frequency and a 200 ms continuous pulse. Blue shading indicates laser pulse timing. **(c)** Trial-avaraged stimulation-onset aligned firing rate for the same stimulation conditions. Shaded regions indicate standard error of the mean.

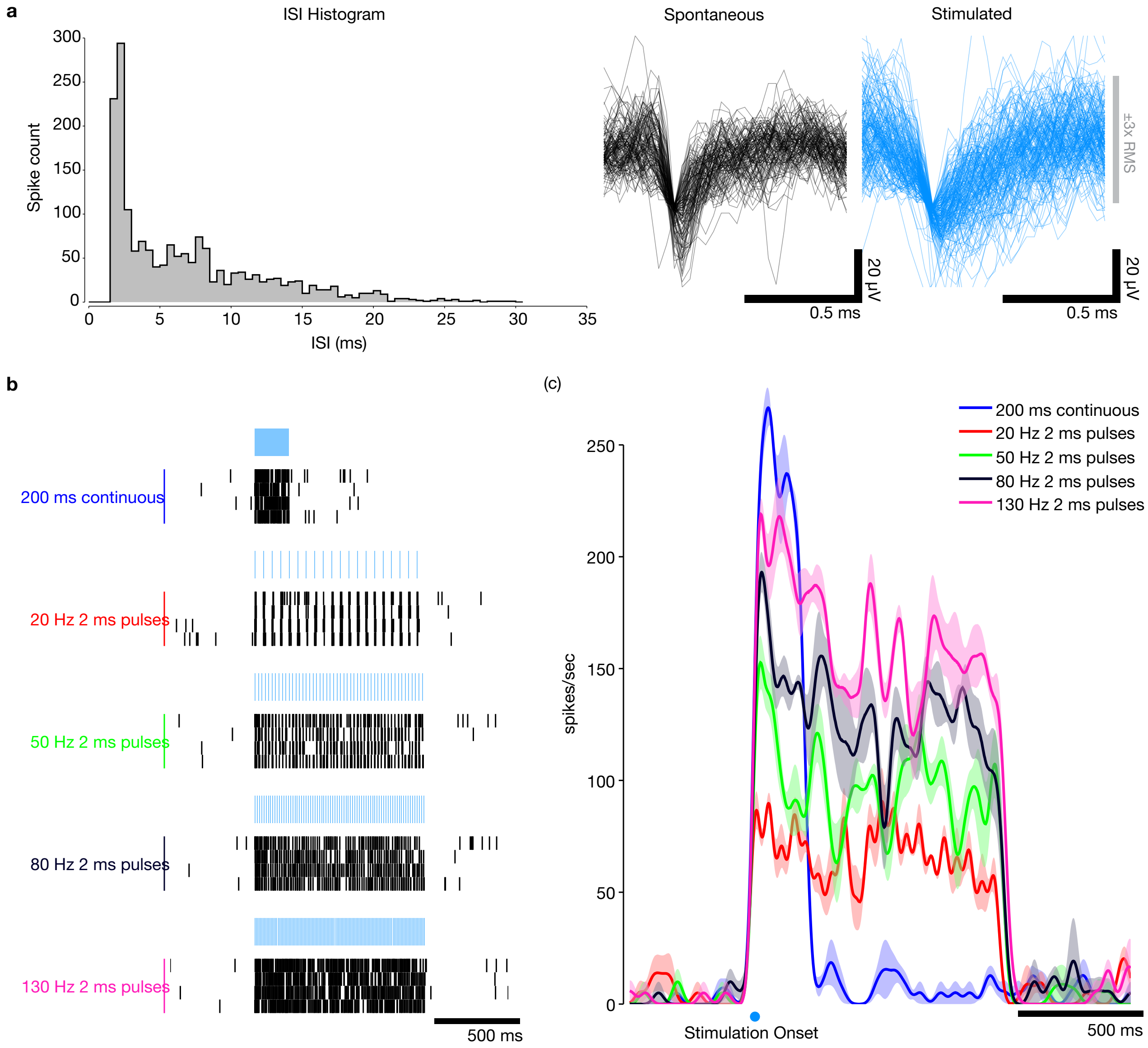

Supp. Figure 7: Response of light-responsive presumed ChR2(H134R)-expressing axons below M1 cortex in Sq. P. This multiunit activity was recorded just below left M1 cortex (3 mm below surface) in Sq. P injected with AAV5-hSyn-ChR2(H134R)-EYFP. (a) Interspike interval histogram and sample spontaneous and light-evoked waveforms recorded for this unit. (b) Stimulation aligned spike raster during 1000 ms pulse trains of varying frequency and a 200 ms continuous pulse. Blue shading indicates laser pulse timing. (c) Trial-avaraged stimulation-onset aligned firing rate for the same stimulation conditions. Shaded regions indicate standard error of the mean.

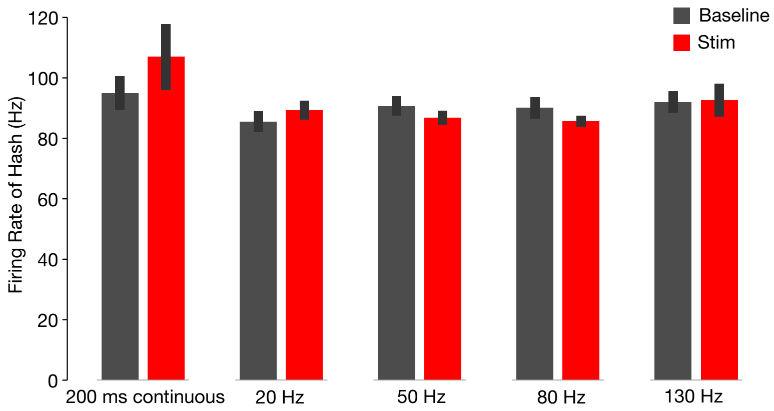

Supp. Figure 8: Response of neural hash to off-wavelength control stimulation in AAV5-hSyn-ChR2(H134R) expressing Sq. P. This low amplitude neural hash (i.e. employing a very liberal threshold for spike detection to capture spiking responses throughout the local population of cells) was recorded in Sq. P left M1, very near the same location as the responsive multi-unit in Supp. Fig. 6. Bars show time-averaged spike rate during stimulation with 3 mW 635 nm light (red) compared to a pre-stimulation window of the same duration (gray). Error bars indicate ± SEM. No significant difference in mean firing rate was detected for any pairwise comparison ($p > 0.3$ in all cases, two-sided sign-rank test).

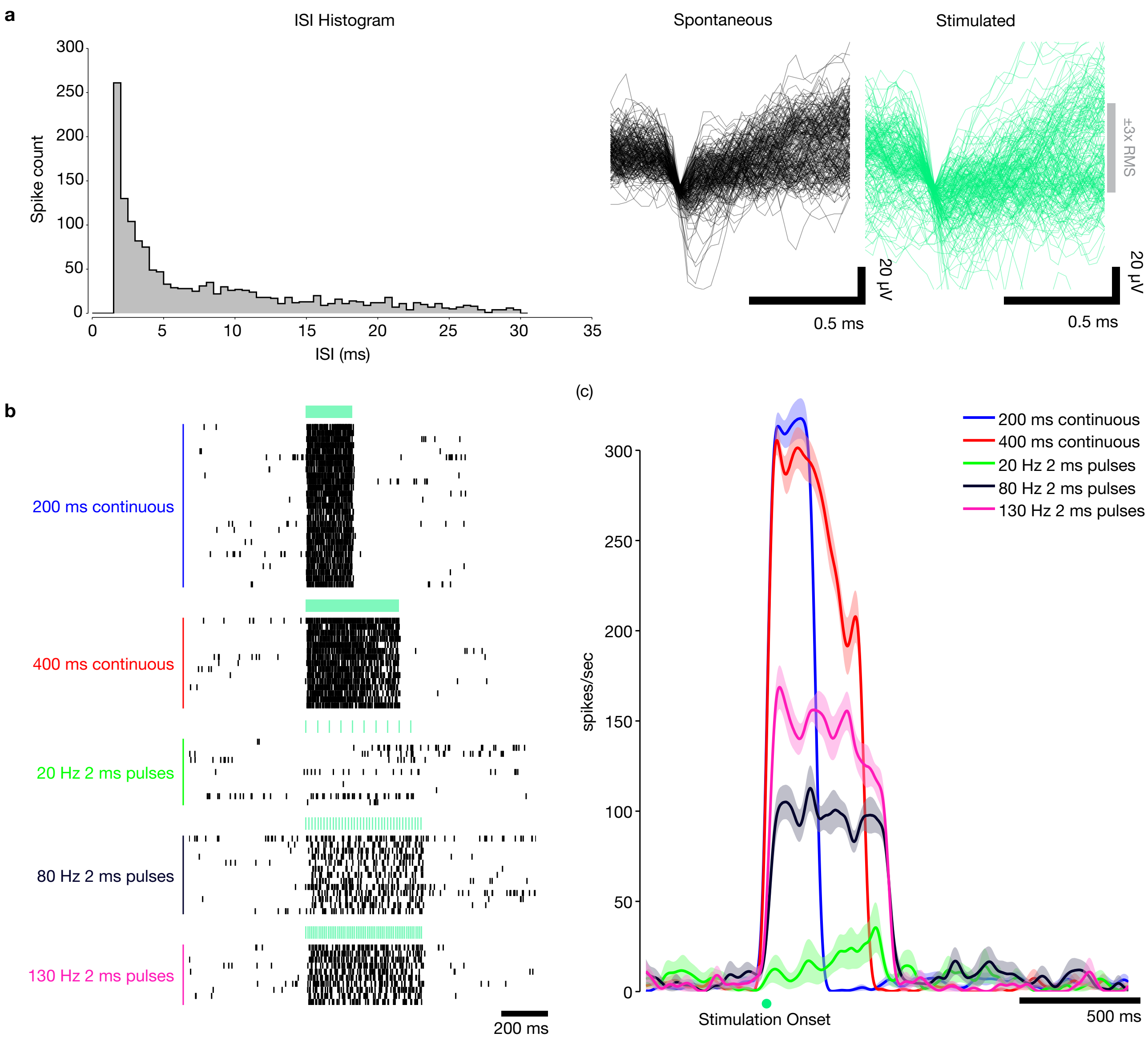

Supp. Figure 9: Response of light-responsive multiunit activity near AAV5-CaMKIIα-C1V1 injection in Sq. U. This multiunit activity was recorded just below left dorsal premotor cortex (PMd) in a monkey injected with AAV5-CaMKIIα-C1V1-EYFP. (a) Interspike interval histogram and sample spontaneous and light-evoked waveforms recorded for this unit. (b) Stimulation aligned spike raster during 1000 ms pulse trains of varying frequency and a 200 ms and 400 ms continuous pulse. Green shading indicates laser pulse timing. (c) Trial-avaraged stimulation-onset aligned firing rate for the same stimulation conditions. Shaded regions indicate standard error of the mean.

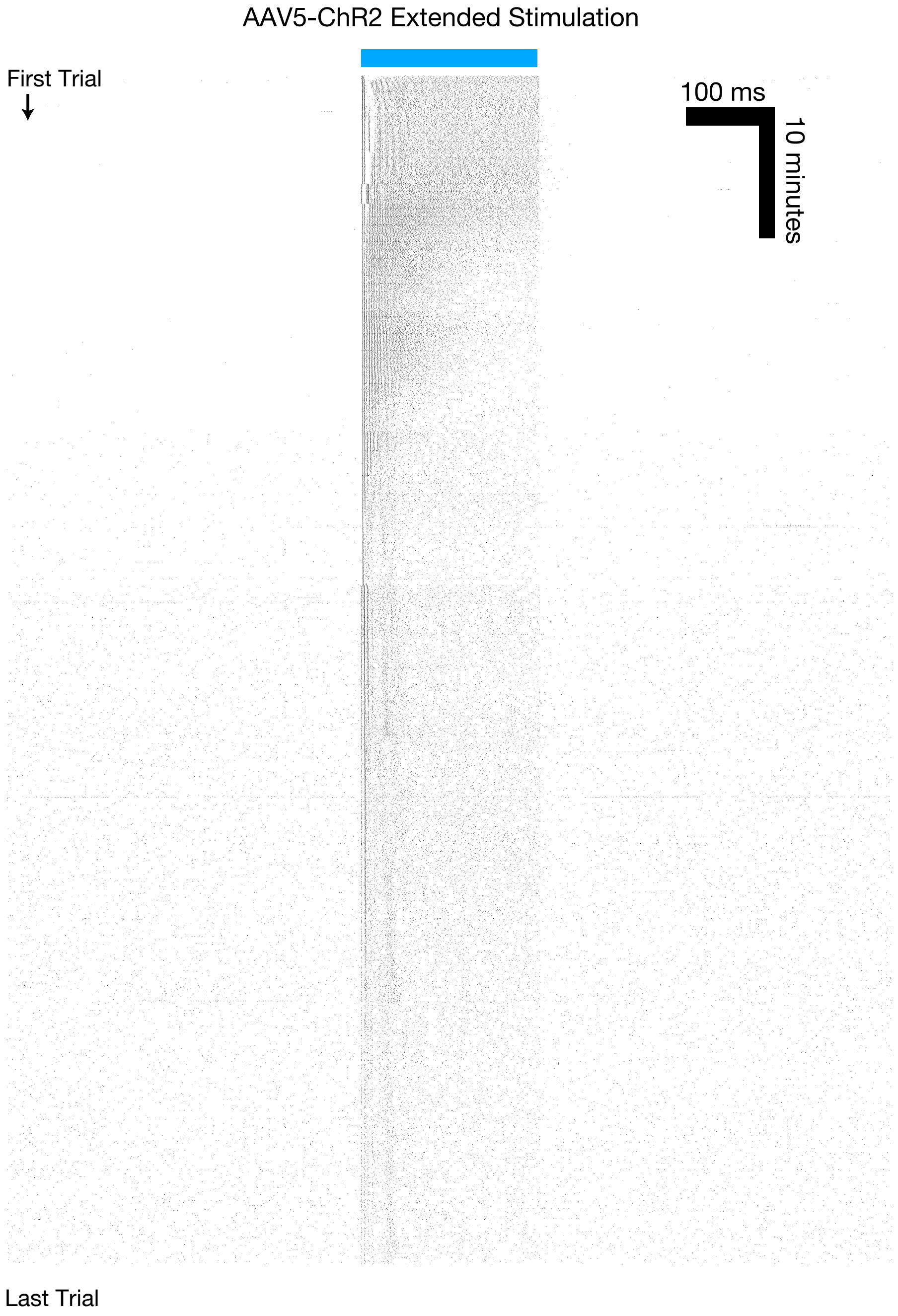

Supp. Figure 10: Stimulation aligned spike raster of multiunit activity recorded in Sq. E during extended stimulation with continuous 200 ms pulses. This multiunit was recorded from left M1 cortex of squirrel monkey Sq. E. injected with AAV5-hSyn-ChR2-EYFP. Stimulation pulses were delivered once per second for 90 minutes. Blue shading indicates laser pulse times.

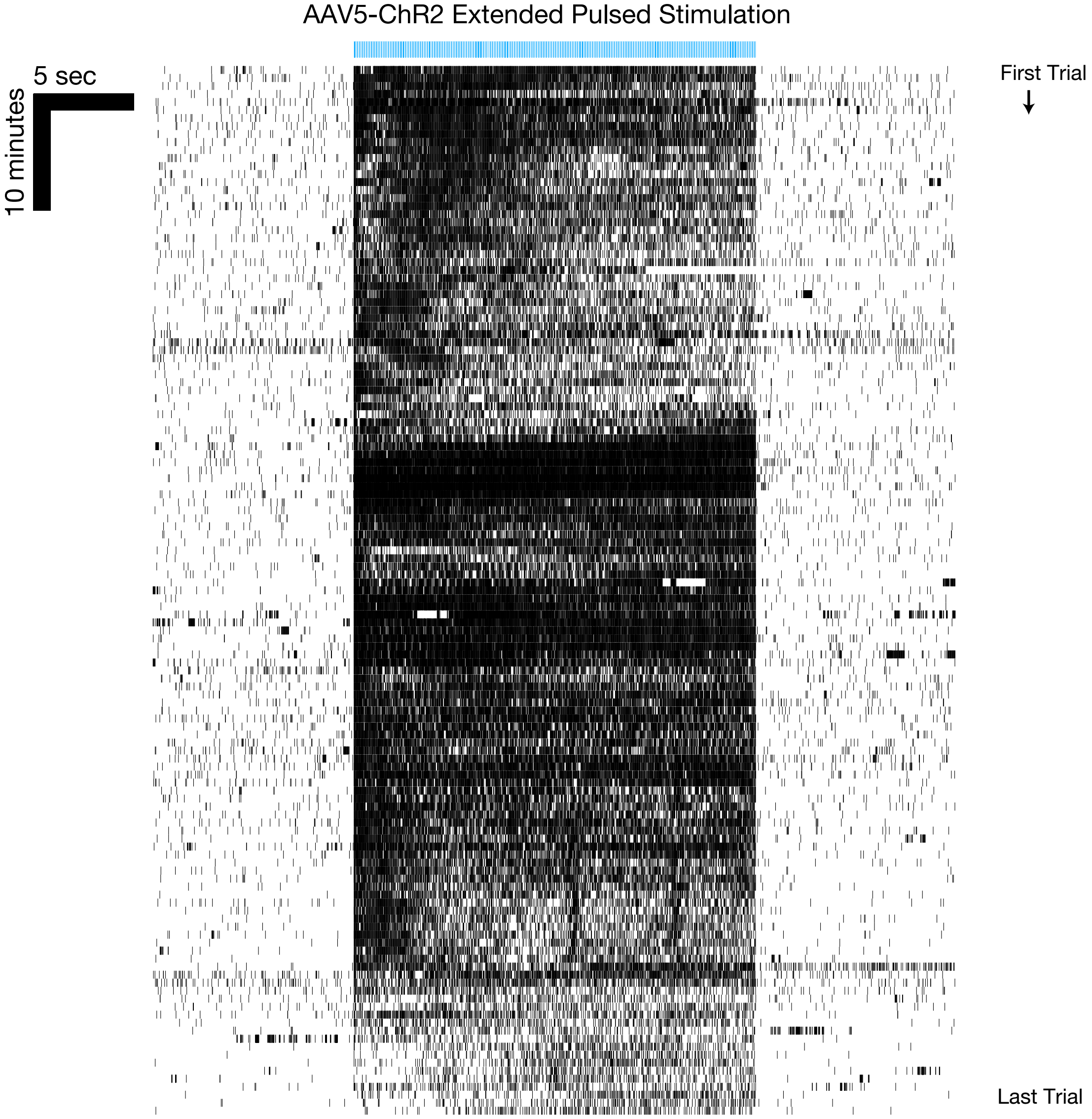

Supp. Figure 11: Stimulation aligned spike raster of multiunit activity recorded during extended stimulation with 50 Hz pulsed light. This multiunit was recorded from left M1 cortex of Sq. T injected with AAV5-hSyn-ChR2-EYFP. Stimulation pulse trains lasted 5 seconds and were delivered once every 15 seconds. Blue shading indicates laser pulse times.

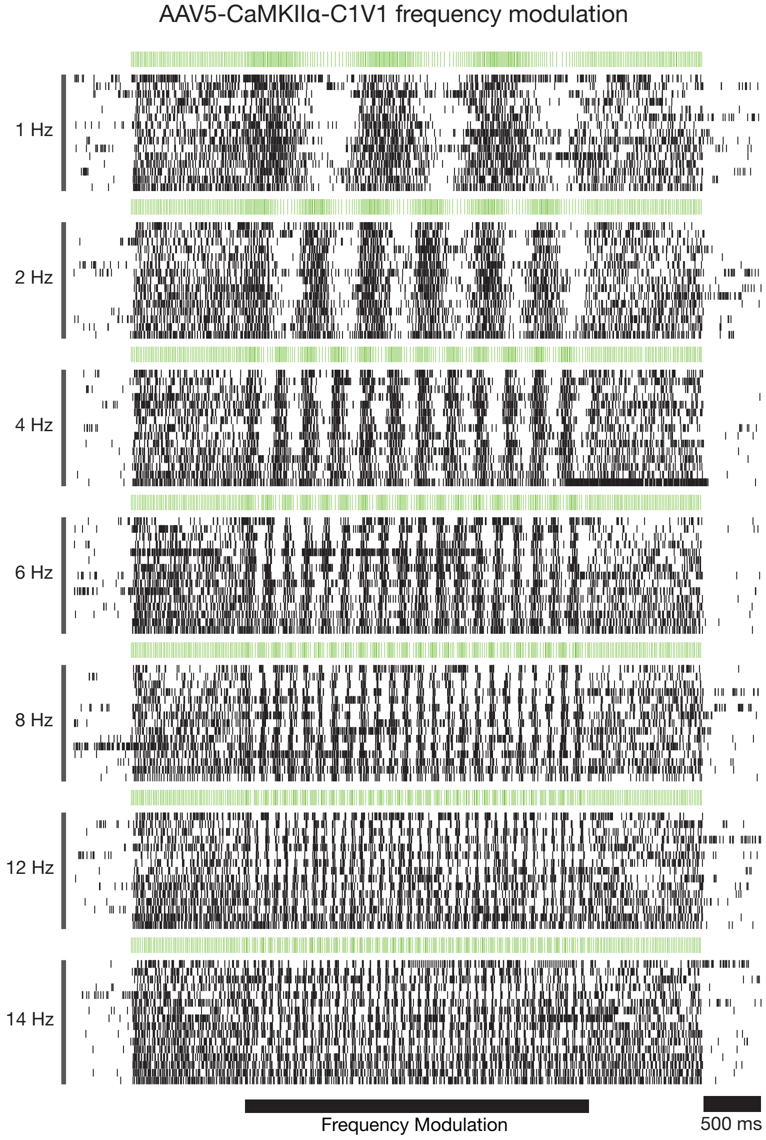

Supp. Figure 12: Spike response raster of an example AAV5-CamKIIα-C1V1 transfected neuron in Sq. U during frequency-modulated pulsed stimulation with 561 nm light. This multiunit activity was recorded in left PMd cortex of Sq. U injected with AAV5-CaMKIIα-C1V1-EYFP. Green horizontal bar indicates overall pulse-train stimulation period. Magenta bar indicates the period of frequency modulation of pulse rate. Labels on the left side indicate the frequency of at which the pulse rate was modulated. Shaded regions indicate standard error of the mean.

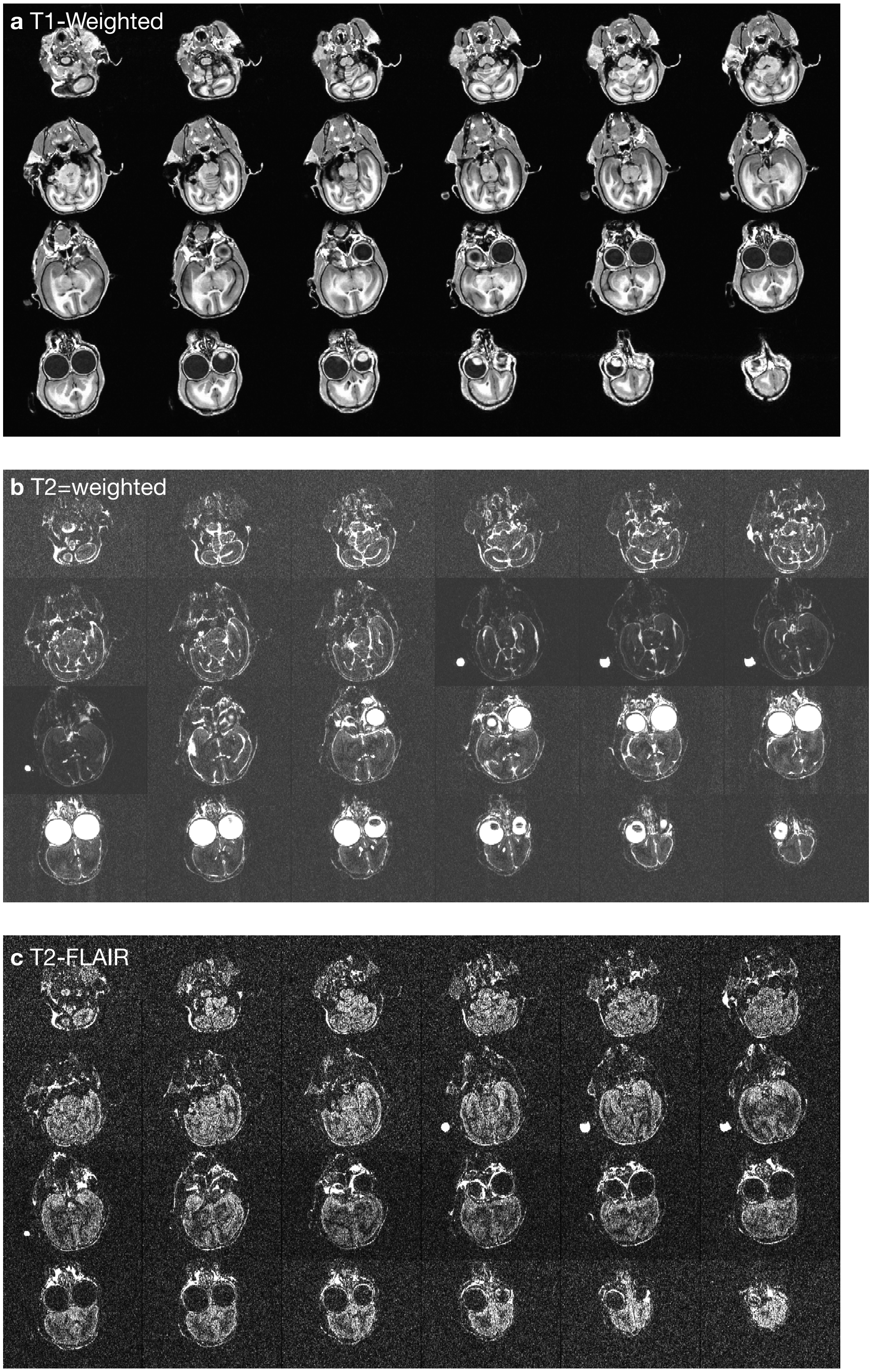
a T1-Weighted
b T2=weighted
c T2-FLAIR

Supp. Figure 13: Long-term axial MR images show normal brain morphology without pathologic changes in Sq. M at one-year post-injection of AAV5-hThy1-eNpHR3.0-EYFP in left M1 and AAV5-CaMKIIα-eNpHR3.0-EYFP in right S1. These images show the three image sequences digitally reformatted in the axial plane as is typically used in clinical presentation. Three types of scans were acquired per monkey: **(a)** T1-weighted a pulse sequence designed to maximize gray vs. white matter contrast), **(b)** T2-weighted, and **(c)** T2-weighted fluid-attenuated inversion recovery (FLAIR), a pulse designed to highlight any pathologic changes. These MR images did not reveal any pathology such as parenchymal injury, hemorrhage, edema, demyelination, or inflammatory changes.

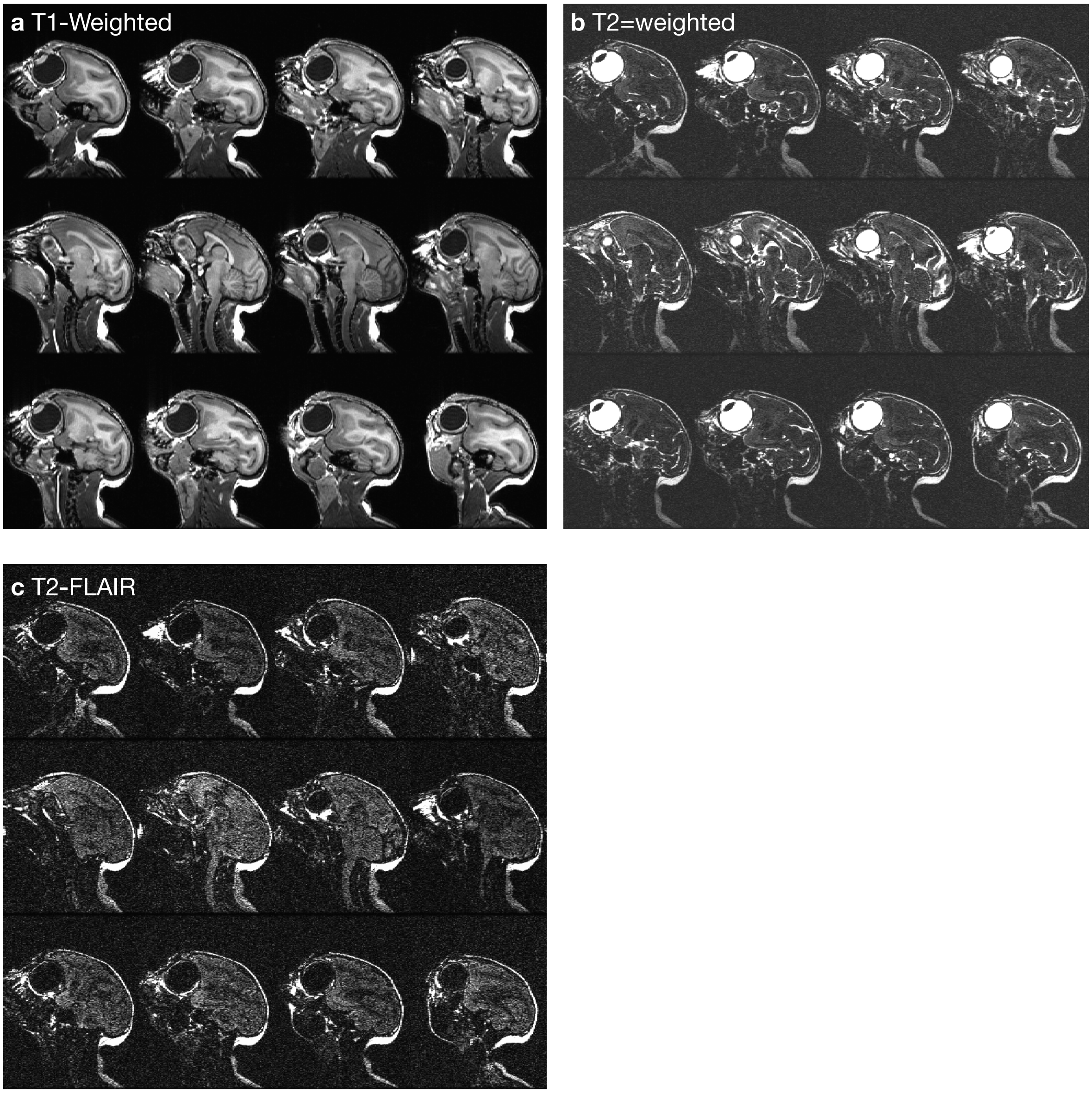

Supp. Figure 14: Long-term sagittal MR images show normal brain morphology without pathologic changes in Sq. O at one-year post-injection of AAV5-hThy1-eNpHR3.0-EYFP in left M1 and AAV5-CaMKIIα-eNpHR3.0-EYFP in right S1. Three types of coronal scans were acquired per monkey and reformatted in the sagittal plane for presentation: (a) T1-weighted a pulse sequence designed to maximize gray vs. white matter contrast), (b) T2-weighted, and (c) T2-weighted fluid-attenuated inversion recovery (FLAIR), a pulse designed to highlight any pathologic changes. These MR images did not reveal any pathology such as parenchymal injury, hemorrhage, edema, demyelination, or inflammatory changes.

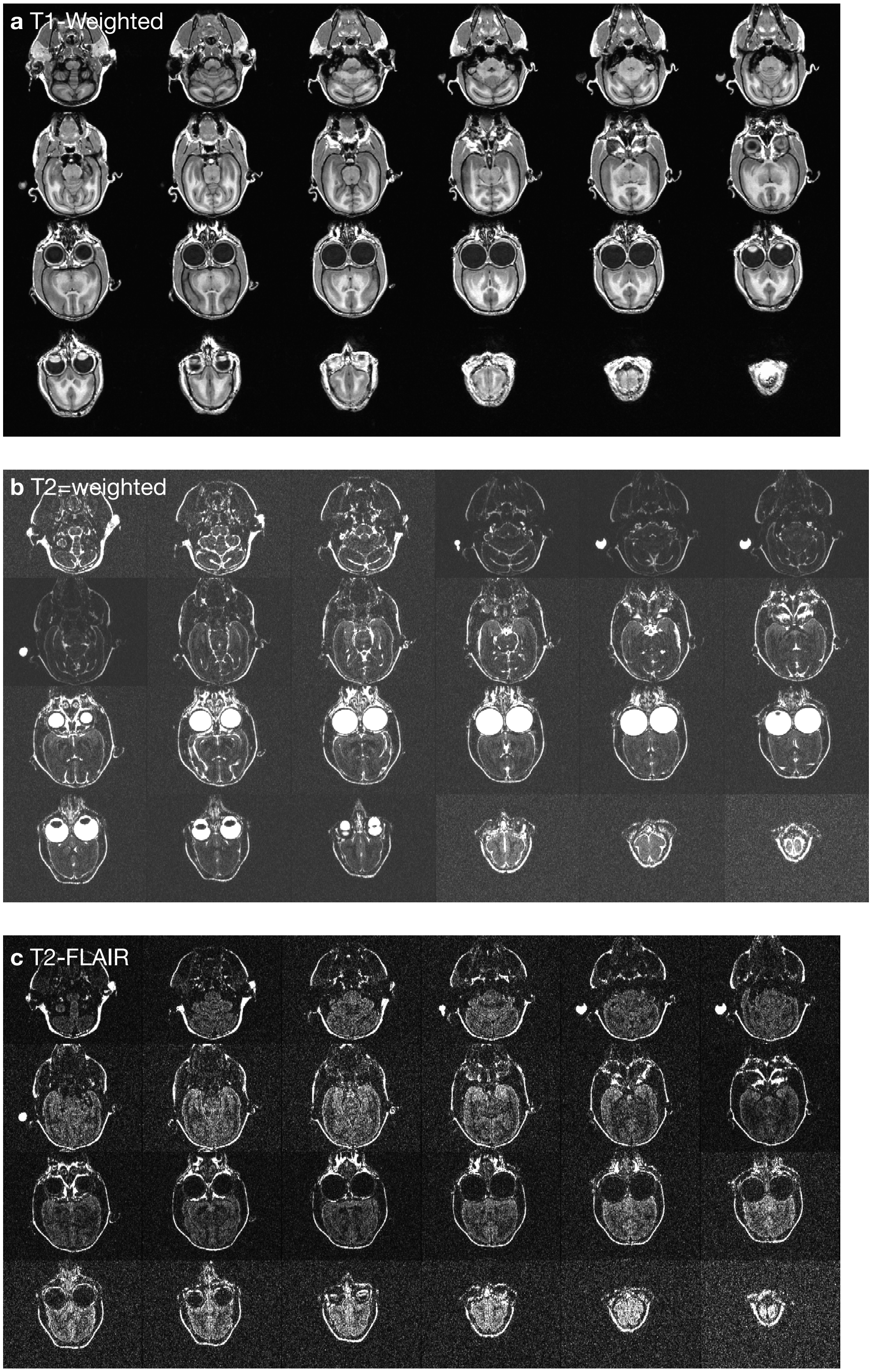
a T1-Weighted
b T2-weighted
c T2-FLAIR

Supp. Figure 15: Long-term axial MR images show normal brain morphology without pathologic changes in squirrel monkey O at one-year post-injection of AAV5-hThy1-eNpHR3.0-EYFP in left M1 and AAV5-CaMKIIα-eNpHR3.0-EYFP in right S1. These images show the three image sequences digitally reformatted in the axial plane as is typically used in clinical presentation. Three types of scans were acquired per monkey: **(a)** T1-weighted a pulse sequence designed to maximize gray vs. white matter contrast), **(b)** T2-weighted, and **(c)** T2-weighted fluid-attenuated inversion recovery (FLAIR), a pulse designed to highlight any pathologic changes. These MR images did not reveal any pathology such as parenchymal injury, hemorrhage, edema, demyelination, or inflammatory changes.

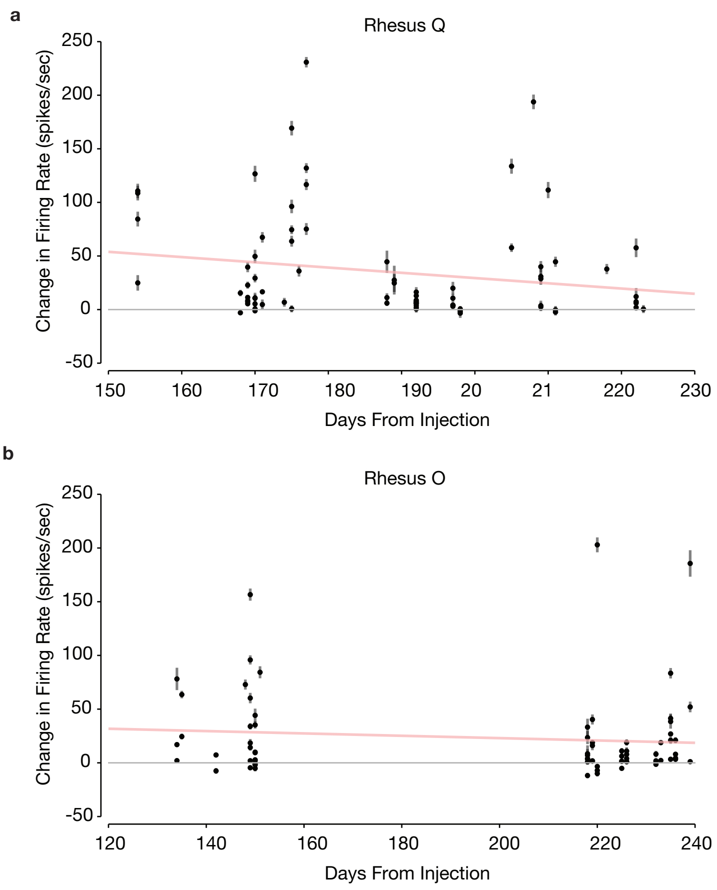

Supp. Figure 16: Optical stimulation reliably elevates firing rates at the recording optrode for many months. Effect of stimulation on firing rate of single or multiunit activity recorded at the stimulating optrode in rhesus macaques Rh. Q (a) and Rh. O (b). Both were injected with AAV5-CaMKIIα-C1V1(TT)-EYFP into PMd. Each dot represents a unit (or multiunit). Vertical axis indicates change in firing rate from interleaved non-stimulated and stimulated time periods during rest or intertrial intervals when no reaching movements were being performed. Vertical bands indicate 95% confidence intervals for the mean change in firing rate. from a t-test. Horizontal axis indicates days from most recent viral injection. Gray horizontal line indicates zero change. Red line indicates linear trend fit to data. For both monkeys, the changes in firing rates vs. days from injection showed a small downward trend that did not reach statistical significance (Rhesus Q: -0.49 spikes/sec/day [-1.08 to 0.10, 95% CI], n=78 units; Rhesus O: -0.11 spikes/sec/day [-0.35 to 0.13, 95% CI], n=74 units).

Supplementary Tables

Construct	Figure panel	Subject	Region Injected	Sterotactic Coordinates (mm)	Depth from dura (mm)	Titer (vg/mL)	Weeks Post Injection
AAV5-CaMKIIα-ChR2(H134R)-eYFP	Figure 1d, Supp. Fig. 1d	Sq. R	Left S1	2 A; 6 L	3,2,1	2x10^{12}	12
AAV5-CaMKIIα-C1V1(TT)-eYFP	Figure 1e, Supp. Fig. 1e	Sq. L	Right Parietal	-2 A; 5 L	3,2,1	2x10^{12}	16
AAV5-CaMKIIα-SFFO-eYFP	Figure 1f, Supp. Fig. 1f	Sq. U	Left S1	2.5, 3.5 A; 7.5 L	3,2	3x10^{12}	19
AAV5-CaMKIIα-eArch3.0-eYFP	Figure 1g, Supp. Fig. 1g	Sq. U	Right S1	2.5 A; 7.5, 8.5 L	3,2	3x10^{12}	19
AAV5-CaMKIIα-eNpHR3.0-eYFP	Figure 1h, Supp. Fig. 1h	Sq. L	Right S1	2 A; 5 L	3,2,1	2x10^{12}	16
AAV5-hSyn-ChR2(H134R)-eYFP	Figure 1i, Supp. Fig. 1i, Figure 5e, Supp. Fig. 11	Sq. T	Left M1	6.5, 7.5 A; 6.5-7.5 L	3,2	1x10^{12}	6
AAV5-hSyn-eNpHR3.0-eYFP	Figure 1j, Supp. Fig. 1j, Figure 2	Sq. B	Left S1	4.5 A; 7.5 L	3,2	3x10^{12}	8
AAV5-hThy1-ChR2(H134R)-eYFP	Figure 1k, Supp. Fig. 1k	Sq. I	Left M1	6.5, 7.5 A; 8.5, 9.5 L	3,2	3x10^{12}	9
AAV5-hSyn-ChR2(H134R)-eYFP	Figure 3a-b, Supp. Fig. 2, Figure 5e, Supp. Fig. 10	Sq. E	Left M1	6.5, 7.5 A; 6.5, 7.5 L	3,2	1x10^{12}	8
AAV5-hSyn-ChR2(H134R)-eYFP	Figure 3c-d	Rh. D	Right M1	7 A; 6 L	1,2,3,4,5,6	1x10^{12}	11
AAV2-Ef1α-mCherry-IRES-WGA-Cre	Figure 4b	Sq. I	Right PMd	12 A; 8.5, 9.5 L	3,2	3x10^{12}	9
AAV8-Ef1α-DIO-ChR2(H134R)-eYFP	Figure 4c	Sq. I	Right M1	10 A; 8.5, 9.5 L	3,2	3x10^{12}	9
AAV5-hSyn-ChR2(H134R)-eYFP	Figure 5a-d, Supp. Fig. 5, Supp. Fig. 6, Supp. Fig. 7, Supp. Fig. 8	Sq. P	Left M1	6.5, 7.5 A; 6.5, 7.5 L	3,2	1x10^{12}	7
AAV5-CaMKIIα-C1V1(TT)-eYFP	Figure 5f, Supp. Fig. 9, Supp. Fig. 12	Sq. U	Left PMd	11 A; 7.5, 8.5 L	3,2	4x10^{12}	19
AAV5-hThy1-ChR2(H134R)-eYFP	Supp. Table 2, Figure 7d-e	Sq. D	Left M1	6.5, 7.5 A; 6.5-7.5 L	3,2	2x10^{12}	63
AAV5-CaMKIIα-ChR2(H134R)-eYFP	Supp. Table 2	Sq. D	Right S1	2.5 A; 7.5, 8.5 L	3,2	2x10^{12}	63
AAV5-hThy1-ChR2(H134R)-eYFP	Supp. Table 2, Figure 7a-c	Sq. H	Left M1	6.5, 7.5 A; 6.5-7.5 L	3,2	2x10^{12}	63
AAV5-CaMKIIα-ChR2(H134R)-eYFP	Supp. Table 2	Sq. H	Right S1	2.5 A; 7.5, 8.5 L	3,2	2x10^{12}	63
AAV5-hThy1-eNpHR3.0-eYFP	Supp. Table 2, Figure 6, Supp. Fig. 13	Sq. M	Left M1	6.5, 7.5 A; 6.5-7.5 L	3,2	2x10^{12}	67
AAV5-CaMKIIα-eNpHR3.0-eYFP	Supp. Table 2, Figure 6, Supp. Fig. 13	Sq. M	Right S1	2.5 A; 7.5, 8.5 L	3,2	2x10^{12}	67
AAV5-hThy1-eNpHR3.0-eYFP	Supp. Table 2, Supp. Fig. 14, Supp. Fig. 15	Sq. O	Left M1	6.5, 7.5 A; 6.5-7.5 L	3,2	2x10^{12}	68
AAV5-CaMKIIα-eNpHR3.0-eYFP	Supp. Table 2, Supp. Fig. 14, Supp. Fig. 15	Sq. O	Right S1	2.5 A; 7.5, 8.5 L	3,2	2x10^{12}	68
AAV5-CaMKIIα-C1V1(TT)-eYFP	Figure 8, Supp. Fig. 16	Rh. Q.	Left PMd	15 A; 14, 15 L	1,2,3,4,5,6	3x10^{12}	188
LT-HSV-Ef1α-Cre-mCherry	Supp. Fig. 3	Sq. R.	Left M1	7 A; 7.5 L	3,2,1	2x10^{12}	12
AAV5-hSyn-DIO-eYFP	Supp. Fig. 3	Sq. R.	Left PMd	11 A; 7.5 L	3,2,1	2x10^{12}	12
AAV5-CaMKIIα-C1V1(TT)-eYFP	Supp. Fig. 16	Rh. O.	Left M1, PMd	14 sites centered at 15 A; 14.5 L	1,2,3,4,5,6	3x10^{12}	n/a

Supp. Table 1: Table of opsin injections. In subject column, Sq. prefix refers to squirrel monkey; Rh. prefix refers to rhesus monkeys. Stereotactic coordinates are listed in mm anterior and lateral of interaural zero. Weeks post injection refers to the date of euthanasia relative to injection.

Construct	AAV5-hThy1-ChR2(H134R)-EYFP				AAV5-hThy1-eNpHR3.0-EYFP					
Subject	Sq. D		Sq. H		Sq. M		Sq. O			
Blood Draw	Pre	Post	Pre	Post	Pre	Post	Pre	Post	Reference Range	Units
Weeks post-injection	-16.9	53.7	-242.7	54.3	-1.9	66.9	-0.7	68		weeks
White Blood Cell	7.79	9.08	5.98	11.9	10.2	7.28	9.40	4.02	4.5 – 17.3	K/μL
Red Blood Cell	7.73	7.53	7.68	7.44	7.66	8.03	7.51	7.88	6.3 – 8.2	M/μL
Hemoglobin	14.5	14.4	14.3	14.2	14.7	14.8	14.8	14.9	12.2 – 15.4	g/dL
Neutrophils	60	33	41	43	56	24	30	15	23 – 80	%
Lymphocytes	25	58	54	42	20	55	50	75	18 – 69	%
Monocytes	3	7	5	9	9	11	10	4	0-7	%
Eosinophils	12	2	n/a	6	15	10	10	6	0 – 21	%
AST	92	142	75.2	131	65	70	112	277	102 – 277	U/L
ALT	100	187	49.3	156	40	59	62	311	116 – 384	U/L
Sodium	166	155	152	148	n/a	149	n/a	148	146 – 160	mmol/L
Potassium	6.4	5.7	3.20	5.9	n/a	3.9	n/a	4.3	4.6 – 7.6	mmol/L
C Reactive Protein	0.00	0.00	n/a	0.00	0.00	0.00	0.00	0.00		mg/L
Tumor Necrosis Factor α	0.00	0.00	n/a	0.00	0.00	0.00	0.00	0.00		pg/L

Supp. Table 2: Table of results from four long term squirrel monkeys. All constructs were injected into M1 on either the left or right hemisphere. Blood was drawn taken before injections (Pre) and at least one year post injection (Post). Although a few mildly out-of-range results were obtained, overall the results suggest that all four monkeys were in good health one year out from the date of injection. Of note is a systematic increase in liver enzymes (AST, ALT) across the four squirrel monkeys. Abbreviations: AST: aspartate aminotransferase, ALT: alanine aminotransferase.
